# Constituents from *Dolichos lablab* L. Flowers and Their Anti-Inflammatory Effects via Inhibition of IL-1β Release

**DOI:** 10.3390/molecules29163751

**Published:** 2024-08-07

**Authors:** Zhongwei Shi, Huimin Li, Jiaming Cheng, Wei Zhang, Jingya Ruan, Qianqian Zhang, Zhunan Dang, Yi Zhang, Tao Wang

**Affiliations:** 1 State Key Laboratory of Component-based Chinese Medicine, Tianjin University of Traditional Chinese Medicine, 10 Poyanghu Road, West Area, Tuanbo New Town, Jinghai District, Tianjin 301617, China; 18322306842@163.com (Z.S.); 15380711687@163.com (H.L.); 2 Tianjin Key Laboratory of TCM Chemistry and Analysis, Tianjin University of Traditional Chinese Medicine, 10 Poyanghu Road, West Area, Tuanbo New Town, Jinghai District, Tianjin 301617, China; c1584172707@163.com (J.C.); zhangwei940905@163.com (W.Z.); ruanjingya@tjutcm.edu.cn (J.R.); qianqian_z0906@163.com (Q.Z.); dangzhunan998@163.com (Z.D.)

**Keywords:** flowers of *Dolichos lablab* L., nitrogenous compounds, terpenoid saponins, IL-1β, anti-inflammation

## Abstract

The occurrence of inflammation is closely related to the activation of the NLRP3 inflammasome. IL-1β produced during the activation of the NLRP3 inflammasome has strong pro-inflammatory activity and can also promote the release of inflammatory factors by other immune cells, exacerbating inflammatory damage to tissues. Utilizing IL-1β as the detection index to find small-molecule inhibitors targeting NLRP3 from natural products will benefit the search for drugs for inflammation-related diseases. During the exploration of anti-inflammatory active components derived from the flowers of *Dolichos lablab* L., an ingredient in traditional Chinese medicine with dual applications in both medicinal treatment and dietary consumption, fourteen compounds (**1**–**14**), including seven previously unreported ones, named flosdolilabnitrogenousols A–D (**1**–**4**) and flosdolilabsaponins A–C (**5**–**7**), were found. Their structures were established through extensive NMR spectra determination, HR-ESI-MS analysis, ECD calculations, and chemical reactions. Flosdolilabsaponin A (**5**) stands out as an exceptionally rare tetracyclic lactone oleane-type saponin. Additionally, the inhibitory activity on IL-1β release of all compounds, without cytotoxicity, was evaluated using BMDMs stimulated with LPS/Nigericin. An Elisa assay revealed that compounds **1**, **8**, **9**, and **11**–**14** exhibited significant inhibition of IL-1β release at a concentration of 30 μM. Structure–activity relationships were also discussed. This study indicates that *D. lablab* flowers possess anti-inflammatory activity, which might exert its effect by suppressing the activation of the NLRP3 inflammasome.

## 1. Introduction

Inflammation is the primary immune response to alert the body to an infection or tissue damage. However, an excessive inflammatory response can lead to ongoing damage and the possibility of developing chronic inflammation [1]. Diseases associated with chronic inflammation have become a major health burden across the world [2]. The occurrence of inflammation is closely related to the activation of the NOD-like receptor family pyrin domain containing 3 (NLRP3) inflammasome. Dysfunction of the NLRP3 inflammasome constitutes an important driving force for inflammation-related diseases such as ulcerative colitis, gout arthritis, atherosclerosis, and type 2 diabetes [3]. Thus, the discovery of NLRP3 inflammasome inhibitors is a very attractive therapeutic strategy for the treatment of inflammation-related diseases.

The activation of the NLRP3 inflammasome mainly includes the priming stage and the assembly stage. Pathogen-associated molecular patterns such as lipopolysaccharides (LPSs) can promote the expression of NLRP3 and Pro-interleukin (IL)-1β through nuclear factor-κB signalling during the priming step [4]. Under the stimulation of damage-associated molecular patterns such as nigericin or adenosine-triphosphate (ATP), the NLRP3 inflammasome activates caspase-1, which subsequently mediates the mutation of IL-1β release and, finally, is involved in the inflammatory response cascade [5]. IL-1β production has strong pro-inflammatory activity and can also encourage other immune cells to release inflammatory factors, exacerbating inflammatory damage to tissues [6]. Therefore, IL-1β, being a key pro-inflammatory cytokine, serves as an essential indicator for the determination of inflammatory activity in vitro. Utilizing IL-1β as the detection index to find small-molecule inhibitors targeting NLRP3 from natural products will benefit the search for drugs for inflammation-related diseases. The NLRP3 inflammasome is predominantly expressed in the cytoplasm of innate immune cells like macrophages [7]. Primary cells such as bone marrow-derived macrophages (BMDMs) exhibit properties and functions that closely resemble those of endogenous macrophages. Thus, LPS/ATP- or LPS/Nigericin-stimulated BMDMs are commonly employed as in vitro models for the preliminary screening of NLRP3 inflammasome inhibitors, taking the production of IL-1β as an indicator.

As an ingredient of traditional Chinese medicine (TCM) with dual applications in both medicinal treatment and dietary consumption, the flowers of *Dolichos lablab* L. (Fabaceae family) are characterized by their neutral, naturally sweet taste and their effects, such as their ability to strengthen the spleen and the stomach, clearing away heat, reducing dampness, and promoting diuresis. They are commonly used to treat dysentery, diarrhea, redness, and leucorrhea, as well as summer heat-related dampness [8]. Pharmacological research shows that a 90% ethanol extract of *D. lablab* flowers can also significantly suppress the expression of inflammatory cytokines such as IL-6, TNF-α, and IL-1β in a mice model of ulcerative colitis and promote the recovery of intestinal epithelial tight junctions [9]. Some reports in the literature suggest that this plant contains volatile components [10], flavonoids [11], and phenolic acids [12]. However, the components responsible for exerting an anti-inflammatory effect are still unclear, hindering the elucidation of this plant’s medicinal effects and mechanism. In this study, a phytochemical investigation was undertaken to elucidate the constituents of *D. lablab* flowers. Furthermore, an Elisa assay on IL-1β was utilized to determine the anti-inflammatory inhibitory effect of the obtained compounds using an LPS/Nigericin-induced BMDM model. Herein, we report the compounds’ isolation, structure elucidation, bioactivities, and structure–activity relationships.

## 2. Results

**Figure 1:**
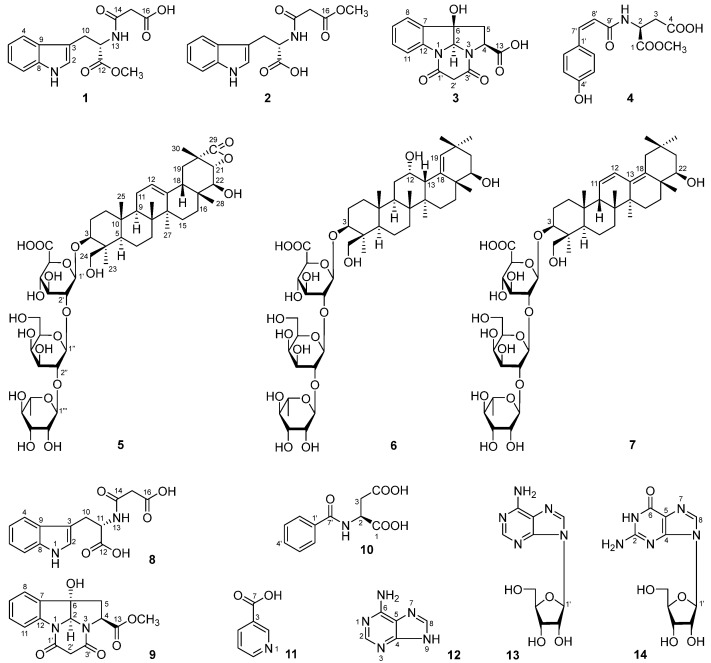
Chemical structures of compounds **1**−**14**.

Our comprehensive phytochemical investigation of *D. lablab* flowers was conducted using silica gel column chromatography (CC), ODS CC, and preparative HPLC (pHPLC) to gain purified compounds. Then, their structures were elucidated by [α]_D_, UV, IR, NMR, and MS spectra, as well as chemical reactions. This effort led to the isolation of seven previously undescribed compounds, flosdolilabnitrogenousols A–D (**1**–**4**) and flosdolilabsaponins A–C (**5**–**7**), along with seven known isolates (**8**–**14**) (Figure 1).

**Figure 2:**
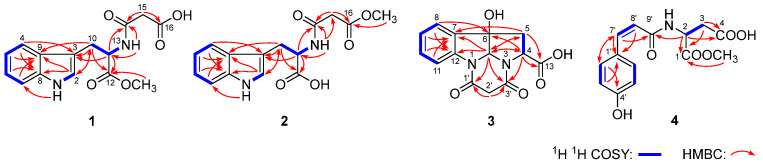
The main ^1^H ^1^H COSY and HMBC correlations of compounds **1**−**4**.

**Figure 3:**
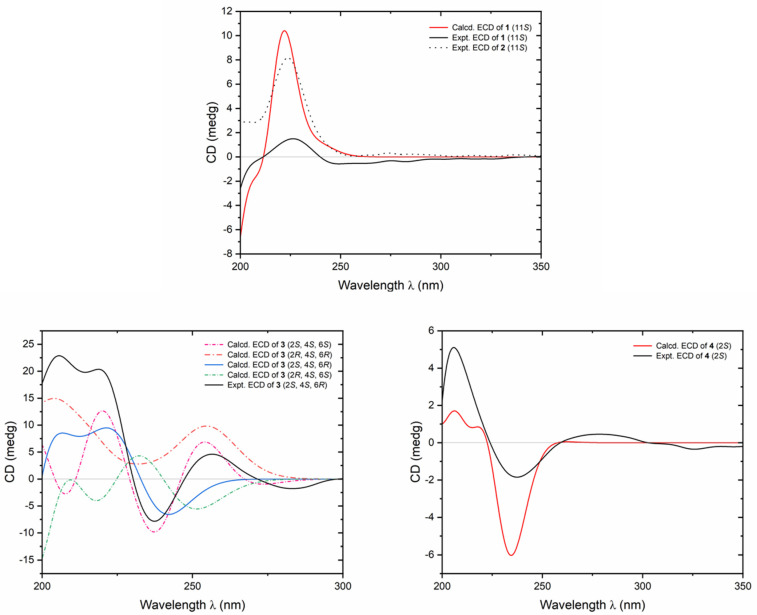
ECD spectra of compounds **1**−**4**.

Flosdolilabnitrogenousol A (**1**) was obtained as a white powder exhibiting positive optical rotation ([α]_D_^25^ +20.8, MeOH). The high-resolution electrospray ionization–mass spectrometry (HR-ESI-MS) analysis revealed an ion peak at *m*/*z* 303.09854 [M − H]^–^ (calculated for C_15_H_15_O_5_N_2_, 303.09755) corresponding to the molecular formula C_15_H_16_O_5_N_2_. Its ^1^H, ^13^C NMR (Table 1), and HSQC spectral determination suggested the presence of one ortho-disubstituted benzene ring [δ_H_ 6.99 (1H, t like, ca. *J* = 8 Hz, H-5), 7.07 (1H, t like, ca. *J* = 8 Hz, H-6), 7.34 (1H, br. d, ca. *J =* 8 Hz, H-7), 7.48 (1H, br. d, ca. *J =* 8 Hz, H-4)], one trisubstituted olefinic bond [δ_H_ 7.16 (1H, d, *J* = 2.5 Hz, H-2)], two methylene {δ_H_ [3.07 (1H, dd, *J* = 7.5, 14.5 Hz), 3.14 (1H, dd, *J* = 5.5, 14.5 Hz), H_2_-10], 3.15 (2H, s, H_2_-15)}, one methine [δ_H_ 4.55 (1H, ddd, *J* = 5.5, 7.5, 7.5 Hz, H-11)], one methoxy [δ_H_ 3.57 (3H, s, 12-COOCH_3_)], two active hydrogen signals [δ_H_ 8.57 (1H, d, *J* = 7.5 Hz, H-13), 10.89 (1H, d, *J* = 2.5 Hz, H-1)], and three ester carbonyl or amide groups [δ_C_ 165.8 (C-14), 169.2 (C-16), 172.0 (C-12)]. The three moieties indicated by bold lines were consolidated by the proton–proton correlations found in its ^1^H ^1^H COSY spectrum (Figure 2). Furthermore, its planar structure was elucidated through the HMBC correlations observed from δ_H_ 10.89 (H-1) to δ_C_ 111.3 (C-7), 127.0 (C-9), 136.0 (C-8); from δ_H_ 7.16 (H-2) to δ_C_ 27.1 (C-10), 127.0 (C-9), 136.0 (C-8); from δ_H_ 7.48 (H-4) to δ_C_ 109.0 (C-3), 136.0 (C-8); from δ_H_ 6.99 (H-5) to δ_C_ 127.0 (C-9); from δ_H_ 7.07 (H-6) to δ_C_ 136.0 (C-8); from δ_H_ 3.07, 3.14 (H_2_-10) to δ_C_ 109.0 (C-3), 123.7 (C-2), 127.0 (C-9), 172.0 (C-12); from δ_H_ 4.55 (H-11) to δ_C_ 109.0 (C-3), 165.8 (C-14), 172.0 (C-12); from δ_H_ 8.57 (H-13) to δ_C_ 53.1 (C-11), 165.8 (C-14); from δ_H_ 3.15 (H_2_-15) to δ_C_ 165.8 (C-14), 169.2 (C-16); and from δ_H_ 3.57 (12-COOCH_3_) to δ_C_ 172.0 (C-12) (Figure 2). Its chemical shifts (Table 1) and optical rotation were closely resembled those of *N*-malonyl-l-tryptophan ([α]_D_^25^ +47.4, MeOH) [13], suggesting that the absolute configuration of C-11 was *S*. This conclusion was supported by the agreement between its calculated and experimental electronic circular dichroism (ECD) curves (Figure 3) [14].

Flosdolilabnitrogenousol B (**2**) was obtained as a white powder with positive optical rotation ([α]_D_^25^ +17.1, MeOH). Its molecular formula, C_15_H_16_O_5_N_2_ (*m*/*z* 303.09842 [M − H]^–^; calculated for C_15_H_15_O_5_N_2_, 303.09755), determined by HR-ESI-MS, was identical to that of compound **1**. Its ^1^H and ^13^C NMR spectra (Table 1) closely resembled those of flosdolilabnitrogenousol A (**1**), with significant differences found in the chemical shifts of C-12, C-14, and C-16 [compound **1**: δ_C_ 165.8 (C-14), 169.2 (C-16), 172.0 (C-12); compound **2**: δ_C_ 164.9 (C-14), 168.1 (C-16), 172.9 (C-12)], indicating the change in the location of methoxy. It was confirmed by the HMBC correlation observed from δ_H_ 3.58 (16-COO*CH*_3_) to δ_C_ 168.1 (C-16) (Figure 2). Ultimately, the absolute configuration of flosdolilabnitrogenousol B (**2**) was elucidated to be 11*S* through a comparison of its experimental ECD spectra with those of compound **1** (Figure 3).

Flosdolilabnitrogenousol C (**3**) was isolated as a yellow powder and showed a pseudomolecular ion peak *m*/*z* 287.06720 [M − H]^–^ (calculated for C_14_H_11_O_5_N_2_, 287.06625) in the HR-ESI-MS analysis. In combination with the ^13^C NMR data, the molecular formula of C_14_H_12_O_5_N_2_ and ten degrees of unsaturation were proposed. Similarly to compound **1**, the ^1^H and ^13^C NMR (Table 2), ^1^H ^1^H COSY, and HSQC spectra suggested the presence of one ortho-disubstituted benzene ring [δ_H_ 7.10 (1H, t like, ca. *J* = 8 Hz, H-9), 7.31 (1H, t like, ca. *J* = 8 Hz, H-10), 7.45 (1H, br. d, ca. *J =* 8 Hz, H-8), 7.68 (1H, br. d, ca. *J =* 8 Hz, H-11)], one “–CH_2_–CH–” moiety {δ_H_ [2.58 (1H, dd, *J* = 2.5, 14.0 Hz), 2.94 (1H, dd, *J* = 10.0, 14.0 Hz), H_2_-5], 4.58 (1H, dd, *J* = 2.5, 10.0 Hz, H-4)}, no other proton-coupled methylene [δ_H_ 2.93, 4.32 (1H each, both d, *J* = 16.0 Hz, H_2_-2′)], and three ester carbonyl or amide groups [δ_C_ 165.9 (C-1′), 167.3 (C-3′), 170.6 (C-13)] in compound **3**. However, comparing its ^1^H and ^13^C NMR spectra with those of **1**, the disappearance of two NH and one olefinic proton signals and the appearance one hydroxyl signal at δ_H_ 6.47 (1H, br. s, 6-OH) and one heteroatom-replaced methine signal at δ_H_ 5.74 (1H, s, H-2), as well as one oxygenated quaternary carbon at δ_C_ 84.4 (C-6), were found. In its HMBC spectrum, the following correlations were observed: from δ_H_ 5.74 (H-2) to δ_C_ 42.7 (C-5), 60.0 (C-4), 133.9 (C-7), 142.1 (C-12), 165.8 (C-1′), 167.3 (C-3′); from δ_H_ 4.58 (H-4) to δ_C_ 83.2 (C-2), 84.4 (C-6), 167.3 (C-3′), 170.6 (C-13); from δ_H_ 2.58, 2.94 (H_2_-5) to δ_C_ 83.2 (C-2), 133.9 (C-7), 170.6 (C-13); from δ_H_ 7.45 (H-8) to δ_C_ 84.4 (C-6), 142.1 (C-12); from δ_H_ 7.10 (H-9) to δ_C_ 133.9 (C-7), 113.9 (C-11); and from δ_H_ 2.93, 4.35 (H_2_-2′) to δ_C_ 165.8 (C-1′), 167.3 (C-3′) (Figure 2). Then, its planar structure was identified, which was the same as that of the reported compound, equisetinine B, with a configuration of 2*S*,4*S*,6*S* [15].

However, their NMR data and optical rotation ([α]_D_^25^ +50.2 for compound **3**; [α]_D_^20^ –89 for equisetinine B, both in MeOH) differed significantly, suggesting distinct configurations between them. Compound **3** possessed three chiral centres—C-2, C-4, and C-6—resulting in eight possible isomers, namely, 2*S*,4*S*,6*S*, 2*R*,4*S*,6*S*, 2*S*,4*S*,6*R*, 2*R*,4*S*,6*R*, and their enantiomers. To elucidate the absolute configuration of compound **3**, the ECD spectra of the 2*S*,4*S*,6*S*, 2*R*,4*S*,6*S*, 2*S*,4*S*,6*R*, 2*R*,4*S*,6*R* isomers were calculated, individually. As a result, the calculated ECD curve of the 2*S*,4*S*,6*R* isomer matched well with the experimental one of compound **3** (Figure 3), suggesting that the absolute configuration of compound **3** was 2*S*,4*S*,6*R* [14]. Consequently, the structure of flosdolilabnitrogenousol C (**3**) was established.

Flosdolilabnitrogenousol D (**4**) was obtained as a white powder with negative optical rotation ([α]_D_^25^ –41.7, MeOH). The HR-ESI-MS analysis revealed its molecular formula as C_14_H_14_O_6_N (*m*/*z* 292.08252 [M − H]^–^; calculated for C_14_H_13_O_6_N, 292.08156). The presence of one *cis*-*p*-coumaroyl [δ_H_ 7.45 (2H, d, *J* = 8.4 Hz, H-2′,6′), 6.72 (2H, d, *J* = 8.4 Hz, H-3′,5′), 6.67 (1H, d, *J* = 12.6 Hz, H-7′), 5.84 (1H, d, *J* = 12.6 Hz, H-8′); δ_C_ 169.9 (C-9′)], one methylene [δ_H_ 2.79 (1H, dd, *J* = 7.2, 17.4 Hz), 2.84 (1H, dd, *J* = 5.4, 17.4 Hz), H_2_-3], one methine [δ_H_ 4.83 (1H, dd, *J* = 5.4, 7.2 Hz, H-2)], one methoxy [δ_H_ 3.73 (3H, s, 1-COO*CH*_3_), and two ester carbonyls [δ_C_ 172.9 (C-1), 174.2 (C-4)] was suggested by its ^1^H, ^13^C NMR (Table 3), and HSQC spectral determination. The above-mentioned fragments were combined to form its planar structure according to the HMBC correlations observed: from δ_H_ 4.83 (H-2) to δ_C_ 169.9 (C-9′), 172.9 (C-1), 174.2 (C-4); from δ_H_ 2.79, 2.84 (H_2_-3) to δ_C_ 172.9 (C-1), 174.2 (C-4); and from δ_H_ 3.73 (1-COO*CH*_3_) to δ_C_ 172.9 (1-COO*CH*_3_) (Figure 2). Its experimental ECD spectra were consistent with the calculated one (Figure 3), suggesting that its absolute configuration was 3*S* [14]. In summary, the structure of flosdolilabnitrogenousol E (**4**) was thus identified.

**Figure 4:**
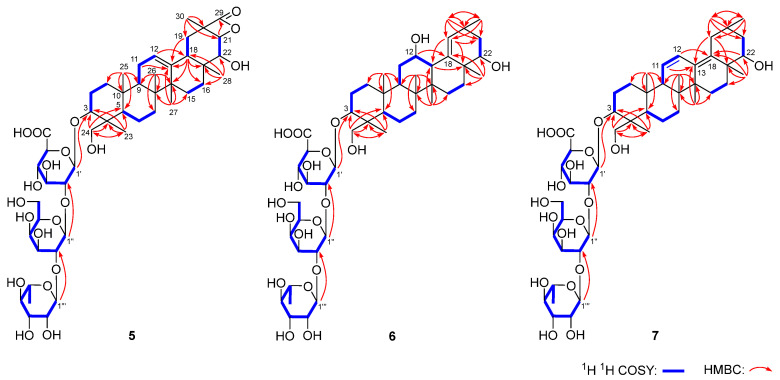
The main ^1^H ^1^H COSY and HMBC correlations of compounds **5**−**7.**

**Figure 5:**
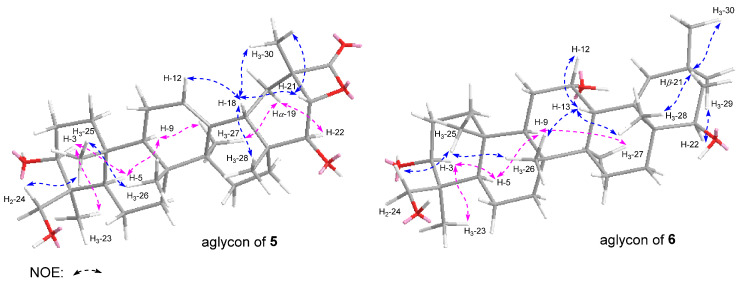
The main NOE correlations of compounds **5** and **6**.

Flosdolilabsaponin A (**5**) was obtained as a white powder with negative optical rotation ([α]_D_^25^ –5.8, MeOH). Its molecular formula was determined to be C_48_H_74_O_20_ (*m*/*z* 969.47211 [M − H]^–^; calculated for C_48_H_73_O_20_, 969.46897) according to the HR-ESI-MS analysis. The characteristic absorptions of the hydroxyl group (3367 cm^–1^), carboxyl (1716 cm^–1^), tetracyclic lactone (1822 cm^–1^), and ether bond (1075 cm^–1^) appeared in the IR spectrum. Flosdolilabsaponin A (**5**) was firstly hydrolyzed with HCl, followed by l-cysteine methyl ester hydrochloride and *O*-toluene isothiocyanate derivatization to obtain its derivative. The comparison of the HPLC retention time of the obtained derivative with that of the derivatives of standard sugar samples indicated the existence of d-glucuronic acid, d-galactose, and l-rhamnose in it [16]. The anomeric proton signals at δ_H_ 5.00 (1H, d, *J* = 7.2 Hz, H-1′), 5.80 (1H, d, *J* = 7.2 Hz, H-1″), and 6.30 (1H, br. s, H-1‴) displayed in its ^1^H NMR spectrum (Table 4) suggested that the three glycosyls were β-d-glucuronopyranosyl, β-d-galactopyranosyl, and α-l-rhamnopyranosyl, respectively. Forty-eight signals were shown in its ^13^C NMR spectrum (Table 4). Except for the eighteen signals which could be assigned to the above three glycosyls, most of the remaining thirty were in the range of δ_C_ 10–60, suggesting that it was terpenoid saponin. Its ^1^H NMR spectrum suggested the presence of six methyl [δ_H_ 0.72, 0.87, 1.13, 1.16, 1.46, 1.57 (3H each, all s, H_3_-25, 26, 27, 28, 23, 29)], one hydroxymethyl [δ_H_ 3.27, 4.28 (1H each, both d, *J =* 11.4 Hz, H_2_-24)], three oxygenated methine [δ_H_ 3.43 (1H, dd, *J =* 3.0, 10.8 Hz, H-3), 4.15 (1H, d, *J =* 7.8 Hz, H-22), 4.57 (1H, d, *J =* 7.8 Hz, H-21)], one olefinic proton [δ_H_ 5.32 (1H, t like, ca. *J* = 3 Hz, H-12)], and one carboxyl group [δ_C_ 175.7 (C-29)], indicating that it was an oleanic acid type of saponin. The nine fragments shown with bold lines were determined by the proton–proton cross-peaks observed in its ^1^H ^1^H COSY spectrum (Figure 4). Furthermore, its planar structure was identified according to the found HMBC correlations: from δ_H_ 1.79 (H-11) to δ_C_ 141.8 (C-13); from δ_H_ 5.32 (H-12) to δ_C_ 47.8 (C-18), 141.8 (C-13); from δ_H_ 1.12, 1.70 (H_2_-15) to δ_C_ 141.8 (C-13); from δ_H_ 2.10 (H-18) to δ_C_ 42.3 (C-14); from δ_H_ 1.13, 2.60 (H_2_-19) to δ_C_ 37.8 (C-17), 141.8 (C-13); from δ_H_ 1.46 (H_3_-23) to δ_C_ 43.9 (C-4), 56.2 (C-5), 63.7 (C-24), 91.2 (C-3); from δ_H_ 3.27, 4.28 (H_2_-24) to δ_C_ 23.1 (C-23), 43.9 (C-4), 56.2 (C-5), 91.2 (C-3); from δ_H_ 0.72 (H_3_-25) to δ_C_ 36.5 (C-10), 38.7 (C-1), 47.8 (C-9), 56.2 (C-5); from δ_H_ 0.87 (H_3_-26) to δ_C_ 33.7 (C-7), 37.8 (C-8), 42.3 (C-14), 47.8 (C-9); from δ_H_ 1.13 (H_3_-27) to δ_C_ 25.8 (C-15), 39.6 (C-8), 42.3 (C-14), 141.8 (C-13); from δ_H_ 1.16 (H_3_-28) to δ_C_ 31.9 (C-16), 37.8 (C-17), 47.8 (C-18), 79.4 (C-22); from δ_H_ 1.57 (H_3_-30) to δ_C_ 35.7 (C-19), 55.4 (C-20), 83.1 (C-21), 175.7 (C-29); from δ_H_ 4.57 (H-21) to δ_C_ 175.7 (C-29); from δ_H_ 5.00 (H-1′) to δ_C_ 91.2 (C-3); from δ_H_ 5.80 (H-1″) to δ_C_ 76.8 (C-2′); and from δ_H_ 6.30 (H-1‴) to δ_C_ 77.8 (C-2″) (Figure 4). Its chemical shifts, C-1–C11 and C-23–C-27, coincided with those of a known compound, soyasaponin Ⅱ [17], indicating that their configurations at C-3–C-5, C-8–C-10, and C-14 were identical. Furthermore, the NOE correlation between δ_H_ 5.32 (H-11) and δ_H_ 2.10 (H-18) suggested that H-18 was β-orientated. Meanwhile, H-21, H_3_-28, and H_3_-30 were also determined to be in a β orientation through the NOE correlations between δ_H_ 2.10 (H-18) and δ_H_ 1.16 (H_3_-28), 1.57 (H_3_-30), and 4.57 (H-21) (Figure 5). Moreover, the coupling constant between δ_H_ 2.60 (H-19α) and δ_H_ 2.10 (H-18) was *J* = 13.2 Hz, suggesting that both H-18 and H-19α were in an axial bond. Furthermore, 22-OH was clarified as having a β orientation according to the NOE cross-peak between δ_H_ 2.60 (H-19α) and δ_H_ 4.15 (H-22). Thus, the structure of flosdolilabsaponin A (**5**) was identified. It is a very rare tetracyclic lactone oleane-type triterpene saponin.

The molecular composition of flosdolilabsaponin B (**6**) was determined to be C_48_H_78_O_19_ (*m*/*z* 957.50568 [M − H]^–^; calculated for C_48_H_75_O_19_, 957.50536). Its ^1^H, ^13^C NMR (Table 4), and 2D NMR spectra, including ^1^H ^1^H COSY, HSQC, and HMBC, suggested that compound **6** owned the same glycosyl, α-l-rhamnopyranosyl(1→2)-β-d-galactopyranosyl(1→2)-β-d-glucuronopyranosyl [δ_H_ 4.97 (1H, d, *J* = 6.6 Hz, H-1′), 5.77 (1H, d, *J* = 7.2 Hz, H-1″), 6.28 (1H, br. s, H-1‴)] as compound **5**. Most of the thirty unattributed carbon signals in the ^13^C NMR spectrum were also in the high field region. Additionally, the signals that could be assigned to seven methyl [δ_H_ 0.70, 1.05, 1.06, 1.17, 1.35, 1.35, 1.41 (3H each, all s, H_3_-25, 26, 29, 30, 27, 28, 23)], one hydroxymethyl [δ_H_ 3.25, 4.26 (1H each, both d, *J =* 11.4 Hz, H_2_-24)], three methine substituted by oxygen [δ_H_ 3.27 (1H, dd, *J =* 4.2, 11.4 Hz, H-3), 4.08 (1H, m, H-22), 4.43 (1H, m, overlapped, H-12)], and one olefinic bond [δ_H_ 5.97 (1H, s, H-19)], present in its ^1^H NMR spectrum, suggested that compound **6** was also an oleanic acid type of saponin. The following HMBC correlations were observed in its HMBC spectrum (Figure 4): from H-12 to C-18; from H-13 to C-17; from H-19 to C-13, C-17, C-21; from H_3_-23 to C-3–C-5, C-24; from H_2_-24 to C-3–C-5, C-23; from H_3_-25 to C-1, C-5, C-9, C-10; from H_3_-26 to C-7–C-9, C-14; from H_3_-27 to C-8, C-13–C-15; from H_3_-28 to C-16–C-18, C-22; from H_3_-29 to C-19–C-21, C-30; and from H_3_-30 to C-19–C-21, C-29. Based on this determination, its planar structure was clarified. By comparing it with compound **5**, it was found that the NMR data of the A ring and A ring substituents of the two compounds were basically same, suggesting that they were consistent in the configuration of the C-3–C-5 and C-8–C-10 positions. The coupling constant between H-12 and H-13 was about zero, indicating that the two were in a *cis*-configuration. Furthermore, the following NOE correlations indicated that H-12, H-13, H_3_-25, H_3_-26, H_3_-28, and 22-OH were all in a β orientation (Figure 5): between δ_H_ 0.70 (H_3_-25) and δ_H_ 1.05 (H_3_-26); between δ_H_ 1.05 (H_3_-26) and δ_H_ 2.40 (H-13); between δ_H_ 2.40 (H-13) and δ_H_ 1.35 (H_3_-28), 4.43 (H-12); between δ_H_ 1.35 (H_3_-28) and δ_H_ 1.97 (Hβ-21); between δ_H_ 1.97 (Hβ-21) and δ_H_ 1.06 (H_3_-30); and between δ_H_ 1.17 (H_3_-29) and δ_H_ 4.08 (H-22). Finally, the position of α-l-rhamnopyranosyl(1→2)-β-d-galactopyranosyl(1→2)-β-d-glucuronopyranosyl’s linkage with aglycon was determined to be position-3 through the HMBC correlation observed from *δ*_H_ 4.97 (H-1′) to δ_C_ 91.3 (C-3).

Flosdolilabsaponin C (**7**) with the molecular formula of C_48_H_76_O_18_ (*m*/*z* 941.50995 [M + H]^–^; calculated for C_48_H_77_O_18_, 941.51044) was also obtained as a white powder. The comparison of the NMR data of **7** (Table 4) with those of **5** and **6** indicated that all of them had a trisaccharide moiety, α-l-rhamnopyranosyl(1→2)-β-d-galactopyranosyl(1→2)-β-d-glucuronopyranosyl. In addition, the ^1^H NMR spectrum of compound **7** suggested the existence of seven methyl [δ_H_ 0.71, 0.76, 0.90, 1.03, 1.10, 1.35, 1.46 (3H each, all s, H_3_-25, 26, 30, 29, 27, 28, 23)], one hydroxymethyl [δ_H_ 3.27, 4.24 (1H each, both d, *J =* 11.4 Hz, H_2_-24)], two oxygenated methin [δ_H_ 3.42 (1H, dd, *J =* 4.2, 11.4 Hz, H-3), 3.80 (1H, dd, *J =* 3.6, 11.4 Hz, H-22), and a pair of olefinic protons coupled to one another [δ_H_ 5.56 (1H, br. d, ca. *J =* 10 Hz, H-11), 6.49 (1H, d, *J* = 10.2 Hz, H-12)]. Combined with its ^13^C NMR spectrum (Table 4), flosdolilabsaponin C (**7**) was also deduced to be an oleanic acid type of saponin. Through the proton–proton cross-peaks displayed in its ^1^H ^1^H COSY spectrum, as well as the long-range correlations found in its HMBC spectrum (Figure 4), the planar structure of **7** was concluded. The chemical shifts in its aglycon were highly consistent with those of astraolesaponin F [18], suggesting that the aglycon of flosdolilabsaponin C (**7**) was (3β,4β,22β)-3,22,24-triol oleana-11,13(18)-diene.

Meanwhile, the structures of the known compounds were identified as *N*-malonyl-l-tryptophan (**8**) [13], equisetinine A (**9**) [15], *N*-benzoylaspartate (**10**) [19], nicotinic acid (**11**) [20], adenine (**12**) [21], β-adenosine (**13**) [22], and guanosine (**14**) [23] (Figure 1) via 1/2D-NMR spectroscopy, HR-ESI-MS, and/or [α]_D_, ECD, as well as a comparison with the literature.

**Figure 6:**
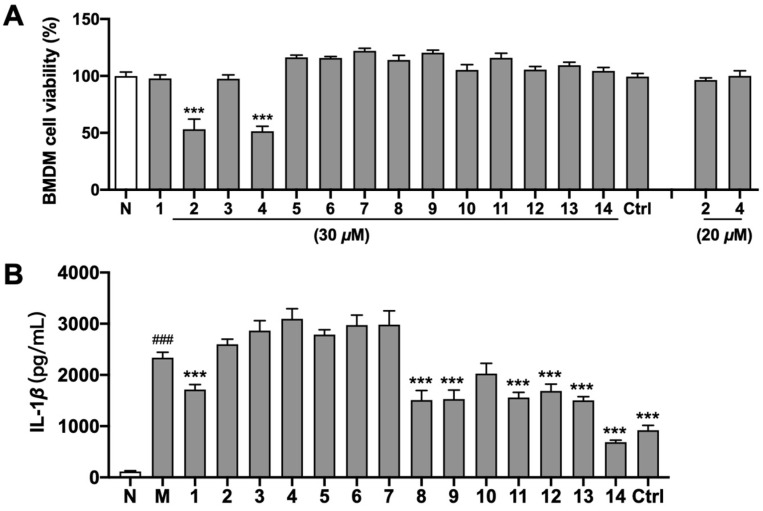
Inhibitory effect of compounds **1–14** on IL-1β release from mice BMDMs. 6A: MTT assay of compounds **1**–**14** on BMDMs from mice. The final administrated concentration of MTT was 500 μg/mL. N: normal group. The values represent the mean ± SD. The drug-treated group vs. N: *** *p* < 0.001. n = 6. 6B: Inhibitory effect of compounds **1**–**14** on IL-1β release from mice BMDMs cells stimulated by LPS/Nigericin. N: normal group; M: model group, 1 μg/mL LPS + 10 μM Nigericin; and Ctrl: MCC950 group. The final administrated concentration of compounds **1**, **3**, and **5**–**14**, as well as MCC950, was 30 μM, while **2** and **4** were 20 μM. The values represent the mean ± SD. M vs. N; ^###^ *p* < 0.001; the drug-treated group vs. M: *** *p* < 0.001. n = 3.

In addition, an LPS/Nigericin-induced BMDM model was used to investigate the inhibitory effects of all the obtained compounds on IL-1β release using inflammasome inhibitor MCC950 as the control group. 3-(4,5-Dimethylthiazol-2-yl)-2,5-diphenyltetrazolium bromide (MTT) experiments were initially conducted to determine the non-cytotoxic concentrations for the bioactivity assay. As shown in Figure 6A, MCC950 and all the compounds, apart from **2** and **4**, exhibited no cytotoxicity on BMDMs at a concentration of 30 μM; when the concentration was reduced to 20 μM, compounds **2** and **4** were eventually safe for the cells. Based on the MTT results, a further Elisa assay for screening the inhibitory effect on IL-1β release suggested that MCC950 as well as compounds **1**, **8**, **9**, and **11**–**14** could effectively reduce the increase in IL-1β secretion in the cell supernatant induced by LPS/Nigericin at the measured concentration. Among the compounds identified from *D. lablab* flowers, **14** was the most effective, and it was stronger than that of the inflammasome inhibitor MCC950 (Figure 6B).

## 3. Discussion

Chronic inflammation plays a significant role in the development of various diseases, such as cancer, cardiovascular disease, diabetes, obesity, autoimmune diseases, and inflammatory bowel disease. Its occurrence is closely related to the activation of the NLRP3 inflammasome [3]. The IL-1β produced during the activation of the NLRP3 inflammasome has strong pro-inflammatory activity and can also promote the release of inflammatory factors by other immune cells, exacerbating inflammatory damage to tissues. Therefore, IL-1β, as a key pro-inflammatory cytokine, is an important indicator for the determination of inflammatory activity in vitro. Currently, the direct or indirect blocking of IL-1β using drugs like anakinra or canakinumab is employed in treating inflammatory diseases [24]. However, canakinumab may lead to side effects such as swelling, diarrhea, and gastroenteritis, while anakinra can also increase infection risk. Medicinal and edible plants with potent therapeutic effects and non-toxic characteristics have attracted more and more researcher attention. As an ingredient in TCM with dual applications in both medicinal treatment and dietary consumption, *D. lablab* flowers have been reported to significantly suppress the expression of inflammatory cytokines such as IL-6, TNF-α, and IL-1β in a mice model of ulcerative colitis [9]. However, the components responsible for exerting this anti-inflammatory effect are still unclear.

Therefore, a combination of various CC and spectral techniques were initially employed to isolate and identify the components from a 70% EtOH extract of *D. lablab* flowers. As results, seven previously undescribed compounds, flosdolilabnitrogenousols A−D (**1**−**4**) and flosdolilabsaponins A−C (**5**−**7**), along with seven known isolates (**8**−**14**) were obtained. Flosdolilabsaponin A (**5**) is an exceptionally rare tetracyclic lactone oleane-type saponin. All the known compounds were first isolated from *Lablab genus*, and equisetinine A (**9**), *N*-benzoylaspartate (**10**), and guanosine (**14**) were first found in the Leguminosae family, as retrieved by Scifinder. This investigation significantly enriches both the chemical composition information database as well as the material database pertaining to *Lablab genus* within the Leguminosae family.

Subsequently, to identify anti-inflammatory active compounds from *D. lablab* flowers, the release of IL-1β from LPS/Nigericin-stimulated mouse BMDM cells was measured using the Elisa method. Notably, flosdolilabnitrogenousol A (**1**), *N*-malonyl-l-tryptophan (**8**), equisetinine A (**9**), nicotinic acid (**11**), adenine (**12**), β-adenosine (**13**), and guanosine (**14**) exhibited significant inhibition of IL-1β release at a concentration of 30 μM. Furthermore, a comparative analysis of nitrogen-containing compounds **1** and **8** suggested that the methyl esterification of 12-carboxyl reduced their IL-1β release inhibitory activity (**1** > **8**). The evaluation of compounds **1**, **2**, and **8** demonstrated that 16-carboxyl was essential for suppressing IL-1β release (**1**, **8** > **2**). Meanwhile, compound configuration greatly influenced the IL-1β release activity of compounds **3** and **9**; notably, the *S*-configured compound exhibited superior activity over the *R*-configured one (**9** > **3**). In addition, the structure–activity relationships (SARs) suggested that the adenine unit was a critical functional group responsible for exerting the inhibitory effect on IL-1β release from LPS/Nigericin-induced BMDMs for **12** and **13**, while d-ribose displayed a negligible impact. These findings suggest that these compounds may exert an anti-inflammatory effect by suppressing the activation of the NLRP3 inflammasome. However, this mechanism remains to be studied.

Moreover, among the obtained compounds, nicotinic acid (**11**) has been previously reported to have anti-inflammatory activity by regulating Sirtuin 1 (SIRT1) expression in LPS/ATP-stimulated human umbilical vein endothelial cells (HUVECs) and reducing reactive oxygen species (ROS) production in HUVECs, thereby inhibiting NLRP3 inflammasome activation and the subsequent caspase-1 cleavage as well as interleukin IL-1β secretion [25]. Additionally, as a key regulator of inflammation, β-adenosine (**13**) has been reported to provide help for many anti-inflammatory drugs such as sulfasalazine and methotrexate, having an anti-inflammatory effect [26]. Meanwhile, guanosine (**14**) has been shown to significantly inhibit the increase in inflammatory factors IL-1, IL-6, TNF-α, and IFN-γ and the decrease in anti-inflammatory factor IL-10 in cerebrospinal fluid after ischemic injury [27]. All the reported information coincides with the experimental results of our research.

Some currently used drugs have no obvious medicinal activity themselves, but they can be used as drug precursors to produce pharmacological components after metabolization in the body. In this study, flosdolilabnitrogenousols B−D (**2**−**4**) and *N*-benzoylaspartate (**10**) showed no significant bioactivity in BMDMs. However, compounds **2** and **3** as well as bioactive compounds **1**, **8**, and **9** might produce malonic acid and the essential amino acid l-tryptophan in vivo. Tryptophan has been reported to enhance the ability of the microbial collective to protect the host against enteric pathogens by activating recombinant dopamine receptor D2 (DRD2) and its downstream pathways, thereby improving gut health and treating severe gastroenteritis, enterocolitis, hemorrhagic diarrhea, and acute kidney failure caused by certain intestinal pathogens [28]. In addition, kynurenine, a metabolite of tryptophan, can reduce acute pancreatitis mortality by inhibiting the production of inflammatory factor TNF-α [29]. Meanwhile, both compounds **4** and **10** might be metabolized to l-aspartic acid in vivo. Aspartate can regulate the activity of inflammasomes through *N*-methyl-d-aspartate (NMDA) receptors and reduce the expression level of inflammasome components and the production of IL-1β [30]. Moreover, as the possible metabolite of compound **4**, coumaric acid, when combined with other anti-inflammatory compounds, could enhance the inhibitory effect on IL-1β release by NLRP3, thereby enhancing the drug’s ability to facilitate recovery from DSS-induced colitis in mice [31].

The above results suggest that *D. lablab* flowers make a very good drug and food dual-use substance, which will play an important role in the prevention and improvement of inflammatory diseases and can be properly consumed in one’s daily diet.

## 4. Experiment

### 4.1. Plant Material

The dried flowers of *D. lablab* were ordered from the Bozhou medicinal material market, Anhui province, China, identified by Professor Lin Ma (Traditional Chinese Medicine of Tianjin University). The specimen (20210920001) was deposited at the Academy of Traditional Chinese Medicine of Tianjin University.

### 4.2. General Experimental Procedures

The NMR spectra were determined on a Bruker ascend 600 MHz and/or 500 MHz NMR spectrometer (Bruker BioSpin AG Industriestrasse 26 CH-8117, Fällanden, Switzerland). The HR-ESI-MS data were acquired on a Thermo HR-ESI-MS mass spectrometer connected to the UltiMate 3000 UHPLC instrument via an ESI interface (Thermo Scientific, Waltham, MA, USA). Optical rotation, UV, IR, and ECD spectral analyses were conducted on a Rudolph Autopol^®^ IV automatic polarimeter (l = 50 mm) (Rudolph Research Analytical, Hackettstown, NJ, USA), a Agilent Cary 60 UV-Vis (Agilent Technologies Inc., Santa Clara, CA, USA), a Varian 640-IR FT-IR spectrophotometer (Varian Australia Pty Ltd., Mulgrave, Australia), and a Circular dichroism spectrum (J-815, JASCO, Tokyo, Japan), respectively. CC isolation was achieved with Macroporous resin D101 (Haiguang Chemical Co., Ltd., Tianjin, China), silica gel (48–75 μm, Qingdao Haiyang Chemical Co., Ltd., Qingdao, China), and ODS (S-50 μm, YMC Co., Ltd., Tokyo, Japan). The HPLC column analysis was performed with Cosmosil 5C_18_-MS-II and a Cosmosil PBr column (4.6 mm i.d. × 250 mm, 5 µm, Nakalai Tesque, Inc., Tokyo, Japan). The compounds were purified with Cosmosil 5C_18_-MS-II and a Cosmosil PBr column (20 mm i.d. × 250 mm, 5 µm, Nakalai Tesque, Inc., Tokyo, Japan). Dichloromethane (CH_2_Cl_2_), methanol (MeOH), acetonitrile (CH_3_CN), acetic acid (HAc), and other reagents (chromatographically pure or analytically pure) were ordered from Tianjin Concord Technology Co., Ltd., Tianjin, China.

### 4.3. Extraction and Isolation

The dried *D. lablab* flowers (10.0 kg) were sequentially extracted with 14 L, 10 L, and 10 L of 70% EtOH for 3 h, 2 h, and 2 h, respectively. After filtration, the solvent was removed under reduced pressure using a rotary evaporator to yield a 70% ethanol extract (4.5 kg), 4.0 kg of which was suspended in H_2_O (6 L) and then partitioned in EtOAc-H_2_O (3 × 6.0 L) to obtain a H_2_O extract (3.6 kg). The H_2_O extract (3.2 kg) was loaded onto D101 macroporous resin CC (H_2_O → 95% EtOH) to gain a H_2_O eluate (2.9 kg) and a 95% EtOH eluate (DLFC, 224.0 g), respectively.

DLFC (150.0 g) was fractioned by silica gel CC [CH_2_Cl_2_-MeOH (100:1 → 20:1 → 100:7 → 10:1 → 7:1 → 5:1 → 4:1 → 3:1 → 2:1 → 0:100, *v*/*v*)] to yield DLFC 1–DLFC 13. DLFC 3 (6.9 g) was further loaded onto ODS CC [MeOH-H_2_O (25:75 → 30:70 → 40:60 → 50: 50 → 60:40 → 70:30 → 80:20 → 90:10 → 100:0, *v*/*v*) to give DLFC 3-1–DLFC 3-10. DLFC 3-1 (515.3 mg) was separated by pHPLC [MeOH-1% HAc (18:82, *v*/*v*), Cosmosil PBr column] to gain DLFC3-1-1–DLFC3-1-4. DLFC 3-1-1 (8.9 mg) was further purified by pHPLC [MeOH-1% HAc (10:90, *v*/*v*), Cosmosil PBr column] to obtain nicotinic acid (**11**, 3.2 mg, *t*_R_ 25.9 min). DLFC 4 (5.5 g) was subjected to ODS CC [MeOH-H_2_O (20:80 → 30:70 → 40:60 → 50:50 → 60:40 → 70:30 → 80:20 → 90:10 → 100:0, *v*/*v*)] to produce DLFC 4-1–DLFC 4-11. DLFC 4-2 (508.2 mg) was prepared by pHPLC [CH_3_CN-1% HAc (8:92, *v*/*v*), Cosmosil 5C_18_-MS-II column] to obtain DLFC 4-2-1–DLFC 4-2-4. Among them, DLFC 4-2-4 was identified as *N*-benzoyl-l-aspartic acid (**10**, 25.1 mg, *t*_R_ 43.2 min). DLFC 4-2-3 (58.1 mg) was purified by pHPLC [MeOH-1% HAc (24:76, *v*/*v*), Cosmosil PBr column] to obtain flosdolilabnitrogenousol C (**3**, 25.8 mg, *t*_R_ 42.1 min). DLFC 4-4 (1.0 g) was separated by pHPLC with CH_3_CN-1% HAc (16:84, *v*/*v*) on a Cosmosil 5C_18_-MS-II column to obtain DLFC 4-4-1–DLFC 4-4-7. DLFC 4-4-5 (121.9 mg) was further prepared by pHPLC [MeOH-CH_3_CN-1% HAc (26:10:64, *v*/*v*/*v*), Cosmosil PBr column] to yield equisetinine A (**9**, 50.2 mg, *t*_R_ 48.4 min). DLFC 5 (3.5 g) was fractioned by ODS CC [MeOH-H_2_O (20:80 → 30:70 → 40:60 → 50: 50 → 60:40 → 70:30 → 80:20 → 90:10 → 100:0, *v*/*v*)] to produce DLFC 5-1–DLFC 5-8. DLFC 5-2 (700.0 mg) was isolated by pHPLC [CH_3_CN-1% HAc (10:90, *v*/*v*), Cosmosil 5C_18_-MS-II column] to obtain DLFC 5-2-1–DLFC 5-2-5. DLFC 5-2-5 (12.2 mg) was further purified by pHPLC [CH_3_CN-1% HAc (15:85, *v*/*v*), Cosmosil PBr column] to gain flosdolilabnitrogenousol D (**4**, 2.3 mg, *t*_R_ 53.8 min). DLFC 5-4 (790.4 mg) was separated by pHPLC [CH_3_CN-1% HAc (22:78, *v*/*v*), Cosmosil 5C_18_-MS-II column] to give DLFC 5-4-1–DLFC 5-4-4. DLFC 5-4-4 was identified as flosdolilabnitrogenousol A (**1**, 37.6 mg, *t*_R_ 48.9 min). DLFC 5-4-3 (81.3 mg) was further purified by pHPLC [CH_3_CN-1% HAc (26:74, *v*/*v*), Cosmosil PBr column] to yield flosdolilabnitrogenousol B (**2**, 50.7 mg, *t*_R_ 40.3 min). DLFC 6 (7.4 g) was loaded onto ODS CC [MeOH-H_2_O (20:80 → 30:70 → 40:60 → 50: 50 → 60:40 → 70:30 → 80:20 → 90:10 → 100:0, *v*/*v*)] to give DLFC 6-1–DLFC 6-12. DLFC 6-5 (624.0 mg) was purified by pHPLC [CH_3_CN-1% HAc (24:76, *v*/*v*), Cosmosil PBr column] to produce *N*-malonyl-l-tryptophan (**8**, 44.8 mg, *t*_R_ 29.0 min). DLFC 7 (11.2 g) was subjected to ODS CC [MeOH-H_2_O (20:80 → 30:70 → 40:60 → 50: 50 → 60:40 → 70:30 → 80:20 → 90:10 → 100:0, *v*/*v*)] to yield DLFC 7-1–DLFC 7-11. DLFC 7-2 (473.3 mg) was prepared by pHPLC [MeOH-1% HAc (10:90, *v*/*v*), Cosmosil PBr column] to gain adenine (**12**, 37.8 mg, *t*_R_ 14.3 min) and β-adenosine (**13**, 157.2 mg, *t*_R_ 52.0 min). DLFC 9 (21.7 g) was loaded onto ODS CC [MeOH-H_2_O (20:80 → 30:70 → 40:60 → 50: 50 → 60:40 → 70:30 → 80:20 → 90:10 → 100:0, *v*/*v*)] to gain DLFC 9-1–DLFC 9-13. DLFC 9-2 (293.4 mg) was prepared by pHPLC [CH_3_CN-1% HAc (5:95, *v*/*v*), Cosmosil PBr column] to yield guanosine (**14**, 10.6 mg, *t*_R_ 30.4 min). DLFC 9-9 (535.9 mg) was separated by pHPLC [CH_3_CN-1% HAc (32:68, *v*/*v*), Cosmosil 5C_18_-MS-II column] to gain DLFC 9-9-1–DLFC 9-9-4. DLFC 9-9-2 (29.3 mg) was further purified by pHPLC [CH_3_CN-1% HAc (38:62, *v*/*v*), Cosmosil PBr column] to obtain flosdolilabsaponin A (**5**, 9.1 mg, *t*_R_ 33.4 min). DLFC 9-9-4 (30.3 mg) was prepared by pHPLC [CH_3_CN-1% HAc (38:62, *v*/*v*), Cosmosil PBr column] to obtain flosdolilabsaponin B (**6**, 15.1 mg, *t*_R_ 35.7 min). DLFC 10 (19.8 g) was subjected to ODS CC [MeOH-H_2_O (20:80 → 30:70 → 40:60 → 50: 50 → 60:40 → 70:30 → 80:20 → 90:10 → 100:0, *v*/*v*)] and pHPLC [MeOH-1% HAc (73:27, *v*/*v*), Cosmosil 5C_18_-MS-II column] to yield DLFC 10-9-1–DLFC 10-9-5. DLFC 10-9-1 (55.8 mg) was purified by pHPLC [CH_3_CN-1% HAc (40:60, *v*/*v*), Cosmosil PBr column] to yield flosdolilabsaponin C (**7**, 6.3 mg, *t*_R_ 44.6 min). The schematic representation of the extraction and separation of compounds **1**–**14** is shown in Figure S52.

### 4.4. Spectral Data of **1**–**7**

#### 4.4.1. Flosdolilabnitrogenousol A (**1**)

White powder; [α]_D_^25^ +20.8 (*conc* 1.49, MeOH); UV *λ*_max_ (MeOH) nm (log *ε*): 221 (4.26), 282 (3.54), 290 (3.49); CD (*conc* 0.001 M, MeOH) mdeg (*λ*_nm_): –0.53 (246), +1.49 (226); IR *ν*_max_ (KBr) cm^–1^: 3272, 3057, 2952, 1736, 1655, 1542, 1437, 1342, 1024; ^1^H NMR (DMSO-*d*_6_, 500 MHz) and ^13^C NMR (DMSO-*d*_6_, 125 MHz) δ: Table 1; and ESI-Q-Orbitrap MS: *m*/*z* 303.09891 [M − H]^–^ (calculated for C_15_H_15_O_5_N_2_, 303.09755).

#### 4.4.2. Flosdolilabnitrogenousol B (**2**)

White powder; [α]_D_^25^ +17.1 (*conc* 1.50, MeOH); UV *λ*_max_ (MeOH) nm (log *ε*): 221 (4.17), 283 (3.39), 290 (3.32); CD (*conc* 0.001 M, MeOH) mdeg (*λ*_nm_): +8.16 (224), +2.84 (201); IR *ν*_max_ (KBr) cm^–1^: 3319, 3056, 1733, 1660, 1539, 1437, 1343, 1022; ^1^H NMR (DMSO-*d*_6_, 500 MHz) and ^13^C NMR (DMSO-*d*_6_, 125 MHz) δ: Table 1; and ESI-Q-Orbitrap MS: *m*/*z* 303.09842 [M − H]^–^ (calculated for C_15_H_15_O_5_N_2_, 303.09755).

#### 4.4.3. Flosdolilabnitrogenousol C (**3**)

White powder; [α]_D_^25^ +50.2 (*conc* 1.29, MeOH); UV *λ*_max_ (MeOH) nm (log *ε*): 205 (4.24), 250 (3.67), 280 (3.08), 288 (2.94); CD (*conc* 0.001 M, MeOH) mdeg (*λ*_nm_): –1.78 (282), +4.60 (257), –7.83 (237), +22.88 (206). IR *ν*_max_ (KBr) cm^–1^: 3266, 2901, 1704, 1684, 1603, 1481, 1419, 1317, 1024; ^1^H NMR (DMSO-*d*_6_, 500 MHz) and ^13^C NMR (DMSO-*d*_6_, 125 MHz) δ: Table 2; and ESI-Q-Orbitrap MS: *m*/*z* 287.06720 [M − H]^–^ (calculated for C_14_H_11_O_5_N_2_, 287.06625).

#### 4.4.4. Flosdolilabnitrogenousol D (**4**)

White powder; [α]_D_^25^ –41.7 (*conc* 0.23, MeOH); UV *λ*_max_ (MeOH) nm (log *ε*): 203 (4.01), 226 (3.79, sh), 292 (3.81); CD (*conc* 0.001 M, MeOH) mdeg (*λ*_nm_): +0.46 (278), –1.85 (237), +5.10 (206); IR *ν*_max_ (KBr) cm^–1^: 3259, 2953, 1734, 1647, 1606, 1515, 1437, 1020; ^1^H NMR (CD_3_OD, 600 MHz) and ^13^C NMR (CD_3_OD, 150 MHz) δ: Table 3; and ESI-Q-Orbitrap MS: *m*/*z* 292.08252 [M − H]^–^ (calculated for C_14_H_13_O_6_N, 292.08156).

#### 4.4.5. Flosdolilabsaponin A (**5**)

White powder; [α]_D_^25^ –5.8 (*conc* 0.45, MeOH); IR *ν*_max_ (KBr) cm^–1^: 3367, 2935, 1822, 1716, 1609, 1452, 1415, 1348, 1306, 1075, 1047; ^1^H NMR (C_5_D_5_N, 600 MHz) and ^13^C NMR (C_5_D_5_N, 150 MHz) δ: Table 4; and ESI-Q-Orbitrap MS: *m*/*z* 969.47211 [M − H]^–^ (calculated for C_48_H_73_O_19_, 969.46897).

#### 4.4.6. Flosdolilabsaponin B (**6**)

White powder; [α]_D_^25^ –13.6 (*conc* 0.47, MeOH); IR *ν*_max_ (KBr) cm^–1^: 3367, 2945, 1724, 1607, 1449, 1417, 1359, 1305, 1074, 1047; ^1^H NMR (C_5_D_5_N, 600 MHz) and ^13^C NMR (C_5_D_5_N, 150 MHz) δ: Table 4; and ESI-Q-Orbitrap MS: *m*/*z* 957.50568 [M − H]^–^ (calculated for C_48_H_77_O_19_, 957.50536).

#### 4.4.7. Flosdolilabsaponin C (**7**)

White powder; [α]_D_^25^ –29.4 (*conc* 0.16, MeOH); IR *ν*_max_ (KBr) cm^–1^: 3368, 2941, 1728, 1609, 1455, 1412, 1384, 1305, 1047; ^1^H NMR (C_5_D_5_N, 600 MHz) and ^13^C NMR (C_5_D_5_N, 150 MHz) δ: Table 4; and ESI-Q-Orbitrap MS: 941.50995 [M + H]^+^ (calculated for C_48_H_77_O_18_, 941.51044).

### 4.5. Determination of the Absolute Configuration of Sugars in Compound **5**

Compound **5** (3.0 mg) was hydrolyzed with HCl and derived with l-cysteine methyl ester hydrochloride and *O*-toluene isothiocyanate sequentially to obtain derivatives, by referring to the literature [16]. The obtained derivatives were then analyzed using the same HPLC analysis conditions reported in the literature. Afterwards, by comparing their retention times with those of the authentic sample, d-glucuronic (*t*_R_: 20.0 min), d-galactose (*t*_R_: 16.5 min), and l-rhamnose (*t*_R_: 31.2 min) were determined to be present in compound **5**.

### 4.6. ECD Calculation

The ECD spectra for the optimized conformers were calculated at the APFD/6-311+g(2d,p) level with a CPCM solvent model in CH_3_OH. The conformational geometry was optimized using density functional theory, followed by time-dependent density functional theory calculations. The calculated ECD spectra of different conformers were simulated with a half-bandwidth of 0.2 eV calculated using the Gaussian 16 software (Gaussian, Inc., Wallingford, CT, USA). The ECD curves were extracted using the GaussView 6.0 software (Gaussian, Inc., Wallingford, CT, USA). Compound-specific ECD curves were weighted according to the Boltzmann distribution after UV correction. Finally, the calculated data were extracted from GaussView and then treated by the OriginPro 2021 software to compare them with the experimental spectral data.

### 4.7. BMDM Preparation

Male C57BL/6 mice aged 6–8 weeks (Beijing vital river laboratory animal technology, Beijing, China) underwent euthanasia via cervical dislocation. The bone marrow from the femurs and the tibiae was flushed three times using a syringe filled with RPMI-1640 medium (Gibco BRL, Santa Clara, CA, USA). The obtained cells were filtered through a sieve with a pore size of 70 μm, and the erythrocytes were removed using a red blood cell lysing buffer. RPMI-1640 complete medium containing 20 ng/mL recombinant mouse macrophage colony-stimulating factor (M-CSF) obtained from Med Chem Express was used to resuspend the cells gently. The cells were then seeded in 24-well plates at a density of 2 × 10^5^ cells/mL and in 96-well plates at a density of 1 × 10^5^ cells/mL. The cultured cells were then placed in an incubator with 5% CO_2_ at 37 °C to continue culturing until the seventh day, and the culture medium was changed every two days.

### 4.8. MTT Assay

The experimental design encompassed two groups: the normal control and the drug treatment cohorts. After the supernatant of BMDM cells cultured for 7 days was discarded, the drug treatment groups were treated with the tested compounds for 18 h, except for the normal group, where it was changed with the complete culture medium. Then, the supernatant was removed, and 100 μL MTT (Sigma-Aldrich, St. Louis, MO, USA) solution (500 μg/mL) was added, further culturing the cells for 4 h. In the end, formazan crystals were dissolved in dimethylsulfoxide and detected in the absorbance of 490 nm through a BioTek Cytation five-cell imaging multi-mode reader (Winooski, VT, USA).

### 4.9. Inhibitory Effect of the Tested Compounds on LPS/Nigericin-Induced IL-1β Release on BMDMs Measured by Mouse IL-1β Elisa Kit

LPS/Nigericin (Sigma-Aldrich, St. Louis, MO, USA) was used to establish an in vitro cellular inflammation model on BMDMs. A normal group (N), an LPS/Nigericin group (M), the tested compounds’ groups, and MCC950 (Med Chem Express, Monmouth Junction, NJ, USA), known as the Ctrl group, were set. To be more specific, except for the normal group, the LPS/Nigericin group, the Ctrl group, and the tested compounds’ groups were stimulated with 1 μg/mL LPS for 4 h. Then, the culture medium was replaced with RPMI-1640 complete medium, containing different compounds (30 μM or 20 μM) and MCC950 (30 μM) for 4 h. Finally, 10 μM nigericin was added to the cell supernatant for 30 min. The cell supernatant was collected, and the IL-1β concentration was measured according to the instructions of the Elisa kit provided by Elabscience Biotechnology Co., Ltd. (Catalog No: E-EL-M0037; Wuhan, China). Specifically, we added 50 μL of the standard or the collected cell supernatant sample to the microplate and incubated it at 37 °C for 90 min. Then, the liquid was discarded, and 50 μL of biotinylated detection Ab working solution was added to each well. After incubating for 60 min at 37 °C, the liquid was removed, and the plate was washed three times. Next, 50 μL of HRP conjugate working solution was added. After incubating for 30 min at 37 °C, the liquid was discarded, and the plate was washed five times. A total of 50 μL of substrate reagent was added, followed by incubation for 15 min at 37 °C. Finally, 25 μL of stop solution was added, and the plate was read at 450 nm by a BioTek Cytation five-cell imaging multi-mode reader (Winooski, VT, USA).

### 4.10. Statistical Analysis

The experimental results were analyzed using GraphPad Prism 8.0 (GraphPad Software, Inc., La Jolla, CA, USA). The values were expressed as the mean ± SD. Comparisons between the two groups were made using a *t*-test, whereas comparisons between multiple groups were performed using a one-way analysis of variance (One-way, ANOVA). A value of * *p* < 0.05 was considered statistically significant.

## 5. Conclusions

In summary, seven previously undescribed compounds along with seven known compounds were identified from *D. lablab* flowers. Among them, compound **5** is a rare tetracyclic lactone oleane-type saponin. This paper enriched the compound library regarding *Lablab*. Moreover, compounds **1**, **8**, **9**, and **11**–**14** were suggested to effectively suppress inflammation by inhibiting IL-1β release using LPS/Nigericin-induced BMDMs. This study establishes a foundation for exploring this plant’s application in preventing and treating inflammation-related diseases, while clarifying its clinical potential as both a medicinal drug and a food supplement. However, further action mechanisms remain to be explored.

## Figures and Tables

**Table 1:** ^1^H (500 MHz) and ^13^C (125 MHz) NMR data for compounds **1** and **2** in DMSO-*d*_6_.

No.	1		2	
	δ_C_	δ_H_ (*J* in Hz)	δ_C_	δ_H_ (*J* in Hz)
1	–	10.89 (d, 2.5, 1H)	–	10.88 (d, 2.0, 1H)
2	123.7	7.16 (d, 2.5, 1H)	123.5	7.14 (d, 2.0, 1H)
3	109.0	–	109.5	–
4	117.9	7.48 (br. d, ca. 8, 1H)	118.1	7.53 (dd, 1.0, 8.0, 1H)
5	118.3	6.99 (t like, ca. 8, 1H)	118.3	6.98 (dt like, ca. 1, 8, 1H)
6	120.9	7.07 (t like, ca. 8, 1H)	120.8	7.08 (dt like, ca. 1, 8, 1H)
7	111.3	7.34 (br. d, ca. 8, 1H)	111.2	7.34 (br. d, ca. 8, 1H)
8	136.0	–	135.9	–
9	127.0	–	127.2	–
10	27.1	3.07 (dd, 7.5, 14.5, 1H)	27.0	3.05 (dd, 7.5, 15.0, 1H)
		3.14 (dd, 5.5, 14.5, 1H)		3.18 (dd, 5.5, 15.0, 1H)
11	53.1	4.55 (ddd, 5.5, 7.5, 7.5, 1H)	53.1	4.50 (ddd, 5.5, 7.5, 8.0, 1H)
12	172.0	–	172.9	–
13	–	8.57 (d, 7.5, 1H)	–	8.43 (d, 8.0, 1H)
14	165.8	–	164.9	–
15	42.1	3.15 (s, 2H)	41.8	3.30 (s, 2H)
16	169.2	–	168.1	–
12-COO*CH*_3_	51.7	3.57 (s, 1H)		
16-COO*CH*_3_			51.6	3.58 (s, 3H)

**Table 2:** ^1^H (500 MHz) and ^13^C (125 MHz) NMR for compound **3** in DMSO-*d*_6_.

No.	δ_C_	δ_H_ (*J* in Hz)	No.	δ_C_	δ_H_ (*J* in Hz)
2	83.2	5.74 (s, 1H)	11	113.9	7.68 (br. d, ca. 8, 1H)
4	60.0	4.58 (dd, 2.5, 10.0, 1H)	12	142.1	–
5	42.7	2.58 (dd, 2.5, 14.0, 1H)	13	170.6	–
		2.94 (dd, 10.0, 14.0, 1H)	1′	165.8	–
6	84.4	–	2′	46.9	2.93 (d, 16.0, 1H)
7	133.9	–			4.35 (d, 16.0, 1H)
8	124.4	7.45 (br. d, ca. 8, 1H)	3′	167.3	–
9	124.2	7.10 (t like, ca. 8, 1H)	6-OH	–	6.47 (br. s, 1H)
10	129.6	7.31 (t like, ca. 8, 1H)	13-COOH		12.72 (br. s, 1H)

**Table 3:** The ^1^H and ^13^C NMR data for compound **4** in CD_3_OD.

No.	δ_C_	δ_H_ (*J* in Hz)	No.	δ_C_	δ_H_ (*J* in Hz)
1	172.9	–	3′,5′	116.0	6.72 (d, 8.4, 2H)
2	50.4	4.83 (dd, 5.4, 7.2, 1H)	4′	159.4	–
3	37.0	2.79 (dd, 7.2, 17.4, 1H)	7′	139.4	6.67 (d, 12.6, 1H)
		2.84 (dd, 5.4, 17.4, 1H)	8′	120.5	5.84 (d, 12.6, 1H)
4	174.2	–	9′	169.9	–
1′	127.8	–	1-COO*CH*_3_	53.0	3.73 (s, 3H)
2′,6′	132.6	7.45 (d, 8.4, 2H)			

**Table 4:** ^1^H and ^13^C NMR data for compounds **5**−**7** in C_5_D_5_N.

No.	5		6		7	
	δ_C_	δ_H_ (*J* in Hz)	δ_C_	δ_H_ (*J* in Hz)	δ_C_	δ_H_ (*J* in Hz)
1	38.7	0.83 (m, 1H)	38.7	0.80 (m, 1H)	37.9	0.85 (m, o, 1H)
		1.39 (m, o, 1H)		1.41 (m, o, 1H)		1.62 (m, o, 1H)
2	26.7	1.88 (m, 1H)	26.7	1.86 (m, o, 1H)	26.6	1.94 (m, o, 1H)
		2.23 (m, 1H)		2.11 (m, 1H)		2.26 (m, o, 1H)
3	91.2	3.43 (dd, 3.0, 10.8)	91.3	3.27 (dd, 4.2, 11.4, 1H)	91.2	3.42 (dd, 4.2, 11.4, 1H)
4	43.9	–	44.0	–	43.9	–
5	56.2	0.86 (br. d, ca. 11, 1H)	56.5	0.87 (br. d, ca. 11, 1H)	55.4	0.87 (m, o, 1H)
6	18.5	1.25 (m, 1H)	18.5	1.25 (m, 1H)	18.6	1.29 (m, o, 1H)
		1.55 (m, o, 1H)		1.55 (m, o, 1H)		1.62 (m, o, 1H)
7	33.7	1.40 (m, o, 1H)	35.2	1.52 (m, 1H)	32.8	1.32 (m, 1H)
		1.44 (m, 1H)				
8	39.6	–	41.1	–	40.5	–
9	47.8	1.51 (dd, 7.2, 10.2, 1H)	45.1	1.99 (dd, 7.2, 12.0, 1H)	54.3	1.94 (br. s, 1H)
10	36.5	–	36.4	–	36.2	–
11	23.9	1.79 (m, o, 1H)	31.7	1.42 (m, 1H)	126.2	5.56 (br. d, ca. 10, 1H)
				1.64 (m, 1H)		
12	124.6	5.32 (t like, ca. 3, 1H)	67.9	4.43 (m, o, 1H)	126.5	6.49 (d, 10.2, 1H)
13	141.8	–	43.1	2.40 (br. s, 1H)	135.2	–
14	42.3	–	43.1	–	42.3	–
15	25.8	1.12 (m, o, 1H)	28.9	1.20 (m, 1H)	24.6	1.13 (m, 1H)
		1.70 (m, 1H)		1.87 (m, 1H)		1.68 (m, 1H)
16	31.9	1.84 (m, 2H)	34.3	1.70 (m, 1H)	33.7	1.85 (m, o, 1H)
				2.24 (m, 1H)		2.26 (m, o, 1H)
17	37.8	–	40.4	–	41.2	–
18	47.8	2.10 (br. d, ca. 13, 1H)	140.3	–	137.9	–
19	35.7	1.13 (m, o, 1H)	130.8	5.97 (s, 1H)	37.8	1.88 (d, 14.4, 1H)
		2.60 (dd, 13.2, 13.2, 1H)				2.49 (d, 14.4, 1H)
20	55.4	–	33.9	–	32.3	–
21	83.1	4.57 (d, 7.8, 1H)	42.3	1.87 (m, o, 1H)	44.8	1.76 (dd, 3.6, 11.4, 1H)
				1.97 (dd, 12.6, 12.6, 1H)		1.84 (m, o, 1H)
22	79.4	4.15 (d, 7.8, 1H)	75.3	4.08 (m, 1H)	76.6	3.80 (dd, 3.6, 11.4, 1H)
23	23.1	1.46 (s, 3H)	22.9	1.41 (s, 3H)	22.9	1.46 (s, 3H)
24	63.7	3.27 (d, 11.4, 1H)	63.6	3.25 (d, 11.4, 1H)	63.3	3.27 (d, 11.4, 1H)
		4.28 (d, 11.4, 1H)		4.26 (d, 11.4, 1H)		4.24 (d, 11.4, 1H)
25	16.1	0.72 (s, 3H)	17.4	0.70 (s, 3H)	18.2	0.71 (s, 3H)
26	17.3	0.87 (s, 3H)	16.4	1.05 (s, 3H)	16.6	0.76 (s, 3H)
27	24.3	1.13 (s, 3H)	20.0	1.35 (s, 3H)	20.3	1.10 (s, 3H)
28	22.8	1.16 (s, 3H)	18.9	1.35 (s, 3H)	18.7	1.35 (s, 3H)
29	175.7	–	30.8	1.17 (s, 3H)	32.6	1.03 (s, 3H)
30	19.4	1.57 (s, 3H)	32.2	1.06 (s, 3H)	25.5	0.90 (s, 3H)
1′	105.5	5.00 (d, 7.2, 1H)	105.5	4.97 (d, 6.6, 1H)	105.6	5.03 (d, 7.2, 1H)
2′	76.8	4.60 (m, o, 1H)	76.7	4.58 (dd, 6.6, 9.0, 1H)	76.8	4.46 (dd, 7.2, 8.4, 1H)
3′	78.6	4.62 (m, o, 1H)	78.5	4.61 (dd, 9.0, 9.6, 1H)	78.6	4.65 (dd, 8.4, 8.4, 1H)
4′	73.9	4.46 (dd, 9.6, 9.6, 1H)	74.0	4.43 (m, o, 1H)	73.9	4.48 (dd, 8.4, 9.6, 1H)
5′	77.6	4.62 (m, o, 1H)	77.6	4.69 (d, 8.4, 1H)	77.6	4.66 (d, 9.6, 1H)
6′	172.7	–	172.8	–	172.6	–
1″	101.8	5.80 (d, 7.2, 1H)	101.8	5.77 (d, 6.6, 1H)	101.8	5.81 (d, 6.6, 1H)
2″	77.8	4.57 (m, o, 1H)	77.9	4.55 (dd, 6.6, 9.0, 1H)	77.9	4.58 (dd, 6.6, 8.4, 1H)
3″	76.7	4.11 (dd, 3.0, 9.0, 1H)	76.7	4.09 (br. d, ca. 9, 1H)	76.7	4.12 (br. d, ca. 8, 1H)
4″	71.2	4.40 (br. d, ca. 3, 1H)	71.2	4.39 (br. s, 1H)	71.2	4.41 (br. s, 1H)
5″	76.5	3.94 (t like, ca. 6, 1H)	76.5	3.93 (t like, ca. 5, 1H)	76.5	3.94 (t like, ca. 5, 1H)
6″	61.6	4.31 (dd, 6.0, 10.8, 1H)	61.6	4.30 (dd, 4.8, 10.2, 1H)	61.6	4.32 (dd, 5.4, 10.8, 1H)
		4.43 (dd, 6.0, 10.8, 1H)		4.41 (dd, 6.0, 10.2, 1H)		4.42 (dd, 5.4, 10.8, 1H)
1‴	102.5	6.30 (br. s, 1H)	102.5	6.28 (br. s, 1H)	102.6	6.31 (br. s, 1H)
2‴	72.5	4.81 (br. s, 1H)	72.2	4.81 (br. s, 1H)	72.5	4.83 (br. s, 1H)
3‴	72.8	4.69 (br. d, ca. 8, 1H)	72.5	4.68 (br. d, ca. 8, 1H)	72.9	4.70 (br. d, ca. 9, 1H)
4‴	74.4	4.35 (dd, 9.0, 9.6, 1H)	74.4	4.32 (dd, 8.4, 9.0, 1H)	74.4	4.36 (dd, 9.0, 10.8, 1H)
5‴	69.5	5.00 (m, 1H)	69.4	4.99 (m, 1H)	69.5	5.02 (m, 1H)
6‴	19.0	1.79 (d, 6.0, 3H)	19.0	1.78 (d, 6.0, 3H)	19.0	1.82 (d, 6.0, 3H)

o: overlapped.

## Data Availability

Data are contained within the article and Supplementary Materials.

## Supplementary Materials

The following supporting information can be downloaded at https://www.mdpi.com/article/10.3390/molecules29163751/s1: supplementary data including the NMR and HRESIMS spectra of compounds **1**–**7** and the physical data of compounds **8**–**14** can be found in the online version.
