# Supplementary material for: Constituents from Dolichos lablab L. Flowers and Their Anti-Inflammatory Effects via Inhibition of IL-1β Release
Source: Molecules. 2024 Aug 7;29(16):3751. doi: 10.3390/molecules29163751 (PMC11357617; doi:10.3390/molecules29163751)
Supplement: Supplementary file 1 [file molecules-29-03751-s001.zip › molecules-3127875-supplementary.pdf]

Supplementary materials

Constituents from the Flowers of *Dolichos lablab* L. and Their Anti-inflammatory Effects *via* Inhibiting IL-1β Release

Zhongwei Shi[1,#], Huimin Li[1,#], Jiaming Cheng[2], Wei Zhang[2], Jingya Ruan[2], Qianqian Zhang[2], Zhunan Dang[2], Yi Zhang[1,2,*], Tao Wang[1,2,*]

1 State Key Laboratory of Component-based Chinese Medicine, Tianjin University of Traditional Chinese Medicine, 10 Poyanghu Road, West Area, Tuanbo New Town, Jinghai District, 301617, Tianjin, China; 18322306842@163.com (Z.-W.S.); 15380711687@163.com (H.-M.L.);

2 Tianjin Key Laboratory of TCM Chemistry and Analysis, Tianjin University of Traditional Chinese Medicine, 10 Poyanghu Road, West Area, Tuanbo New Town, Jinghai District, 301617 Tianjin, China; c1584172707@163.com (J.-M.C.); zhangwei940905@163.com (W.Z.); qianqian_Z0906@163.com (Q.-Q.Z.); dangzhunan998@163.com (Z.-N.D.); ruanjingya@ tjutcm.edu.cn (J.-Y.R.);

# These authors contributed equally to the work;

* Correspondence: zhwwxzh@tjutcm.edu.cn (Y.Z.); wangtao@tjutcm.edu.cn (T.W.); Tel./Fax: +86-22-5959-6168 (T.W.)

Figure S1 1H NMR (DMSO-d6, 500 MHz) spectrum of 1

....4

Figure S2 13C NMR (DMSO-d6, 125 MHz) spectrum of 1

....5

Figure S3 1H 1H COSY spectrum of 1

....5

Figure S4 HSQC spectrum of **1** 5

Figure S5 HMBC spectrum of **1** 6

Figure S6 ESI-Q-Orbitrap-MS spectrum of **1** 6

Figure S7 IR spectrum of **1**

....7

Figure S8 ^{1}H NMR (DMSO-d_6, 500 MHz) spectrum of **2**

....8

Figure S9 ^{13}C NMR (DMSO-d_6, 125 MHz) spectrum of **2**

....8

Figure S10 ^{1}H ^{1}H COSY spectrum of **2**

....9

Figure S11 HSQC spectrum of **2** 9

Figure S12 HMBC spectrum of **2** 10

Figure S13 ESI-Q-Orbitrap-MS spectrum of **2**

....10

Figure S14 IR spectrum of 2

....11

Figure S15 1H NMR (DMSO-d6, 500 MHz) spectrum of 3

....12

Figure S16 13C NMR (DMSO-d6, 125 MHz) spectrum of 3

....12

Figure S17 1H 1H COSY spectrum of 3

....13

Figure S18 HSQC spectrum of **3** 13

Figure S19 HMBC spectrum of **3** 14
Figure S21 ESI-Q-Orbitrap-MS spectrum of 3
....14
Figure S21 IR spectrum of 3 15
Figure S22 1H NMR (CD3OD, 600 MHz) spectrum of 4
....16
Figure S23 13C NMR (CD3OD, 150 MHz) spectrum of 4
....16
Figure S24 1H 1H COSY spectrum of 4
....17
Figure S25 HSQC spectrum of 4 17
Figure S26 HMBC spectrum of 4 18
Figure S27 ESI-Q-Orbitrap-MS spectrum of 4
....18
Figure S28 IR spectrum of 4 19
Figure S29 1H NMR (C5D5N, 600 MHz) spectrum of 5
....20
Figure S30 13C NMR (C5D5N, 150 MHz) spectrum of 5
....20
Figure S31 1H 1H COSY spectrum of 5
....21
Figure S32 HSQC spectrum of 5 21
Figure S33 HMBC spectrum of 5 22
Figure S34 NOESY spectrum of 5 22
Figure S35 ESI-Q-Orbitrap-MS spectrum of 5
....23
Figure S36 IR spectrum of 5
……………………..23
Figure S37 1H NMR (C5D5N, 600 MHz) spectrum of 6
....24

Figure S38 13C NMR (C5D5N, 150 MHz) spectrum of 6

....24

Figure S39 1H 1H COSY spectrum of 6

....25

Figure S40 HSQC spectrum of 6 25

Figure S41 HMBC spectrum of 6 26

Figure S42 NOESY spectrum of 6 26

Figure S43 ESI-Q-Orbitrap-MS spectrum of 6

....27

Figure S44 IR spectrum of 6

………………...27

Figure S45 1H NMR (C5D5N, 600 MHz) spectrum of 7

....28

Figure S46 13C NMR (C5D5N, 150 MHz) spectrum of 7

....28

Figure S47 1H 1H COSY spectrum of 7

....29

Figure S48 HSQC spectrum of 7 29

Figure S49 HMBC spectrum of 7 30

Figure S50 ESI-Q-Orbitrap-MS spectrum of 7

....30

Figure S51 IR spectrum of 7

…………………...31

Figure S52. Schematic representation of extraction and separation of compounds 1–14

...........................32

The physical data of compounds **8–14** ……………………..33

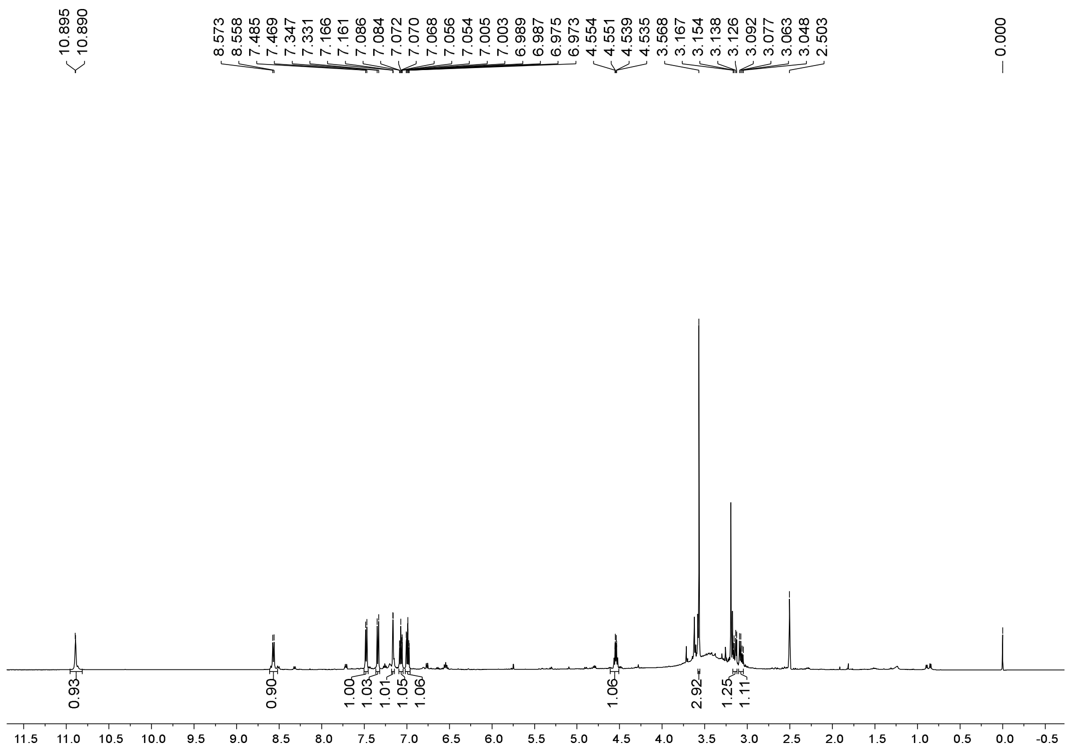

Figure S1. ^{1}H NMR (DMSO-d_6, 500 MHz) spectrum of **1**

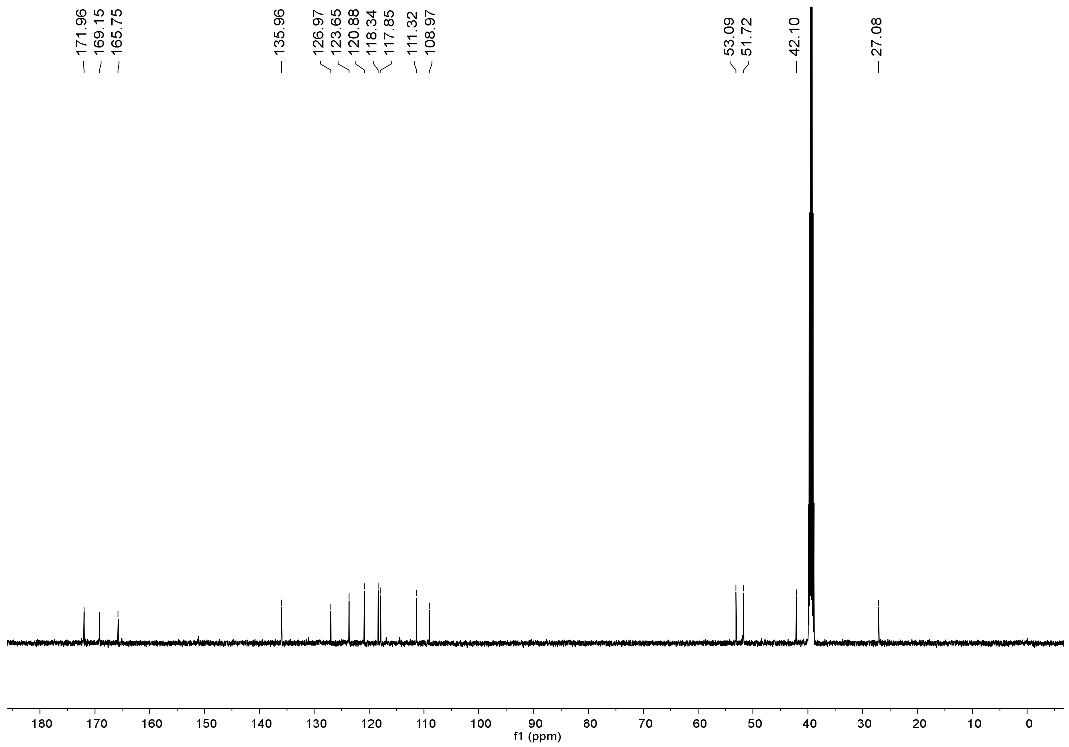

Figure S2. ^{13}C NMR (DMSO-d_6, 125 MHz) spectrum of **1**

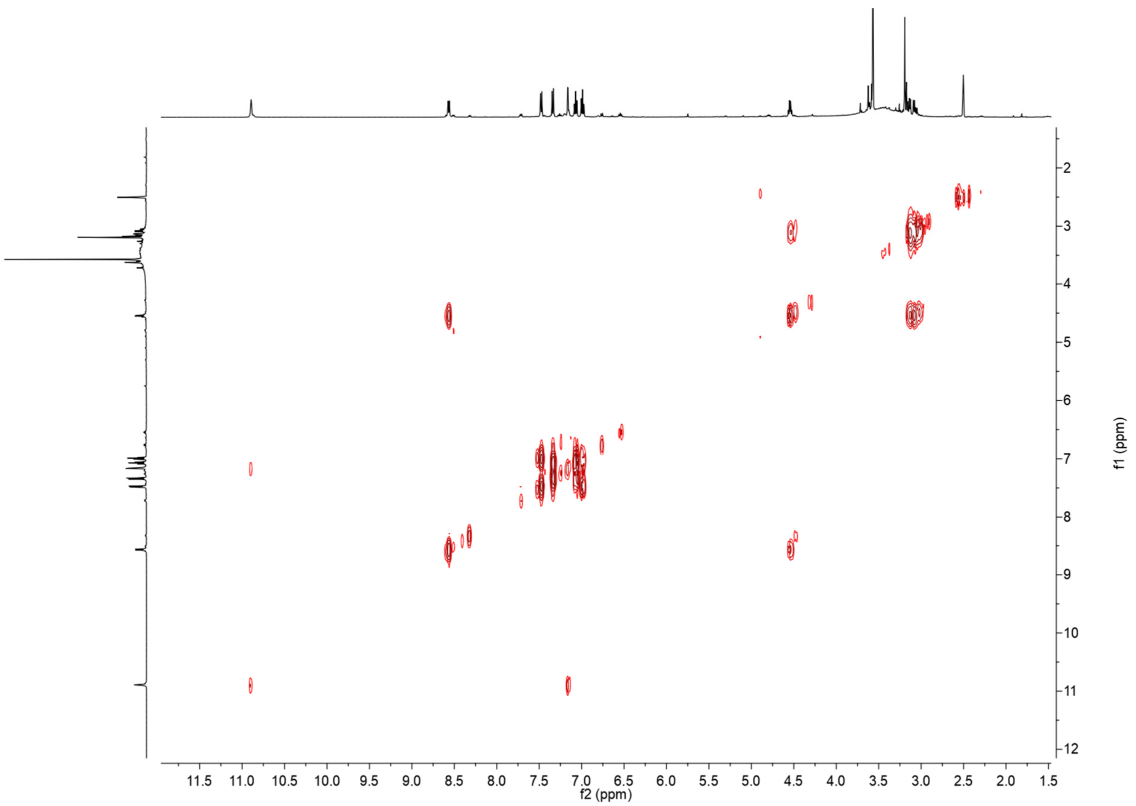

Figure S3. ${}^{1}H$ ${}^{1}H$ COSY spectrum of **1**

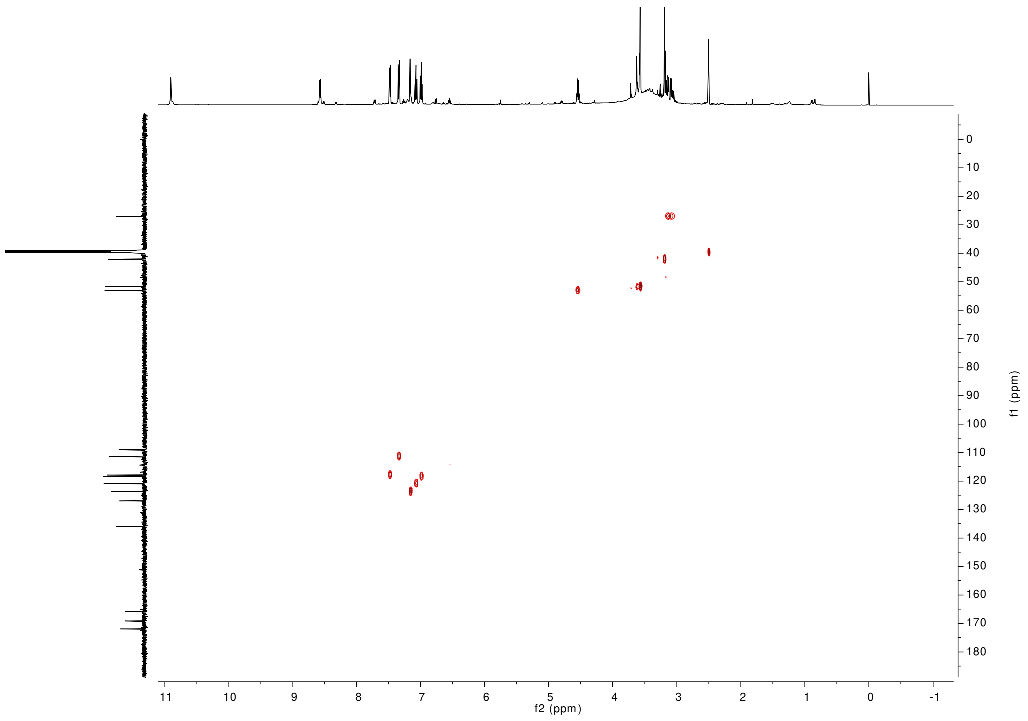

Figure S4. HSQC spectrum of **1**

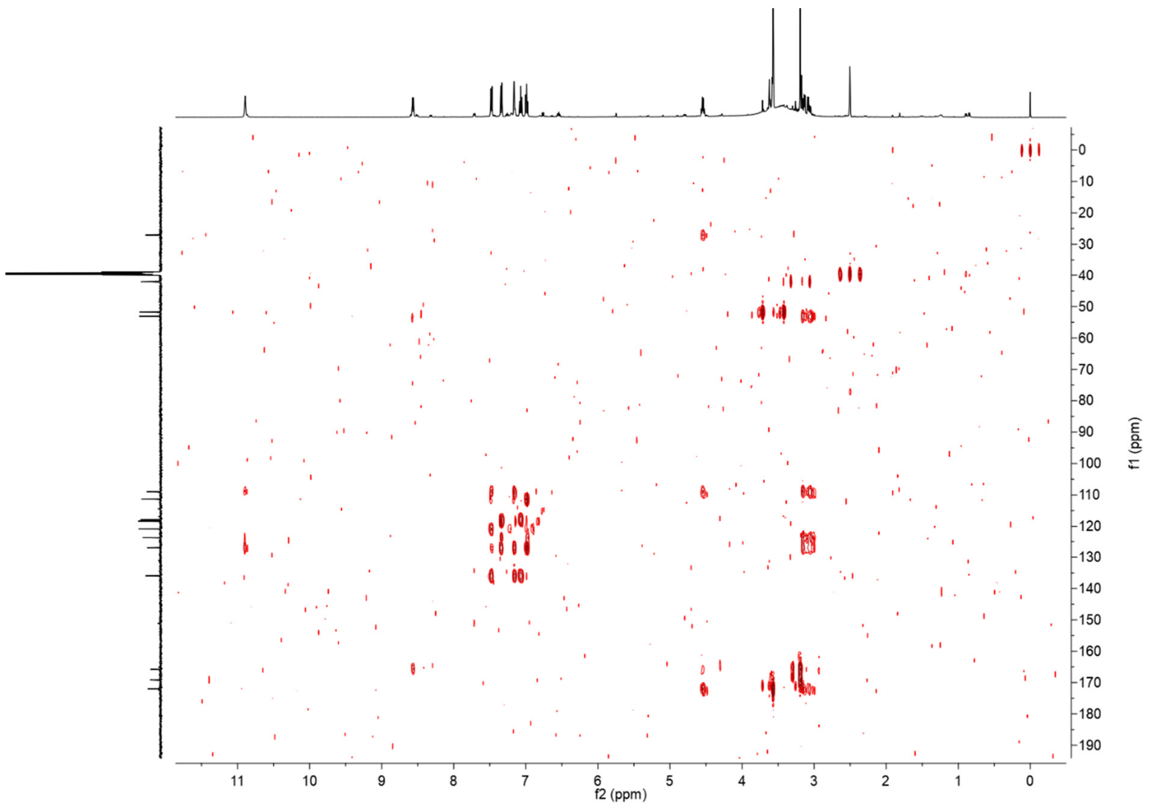

Figure S5. HMBC spectrum of **1**

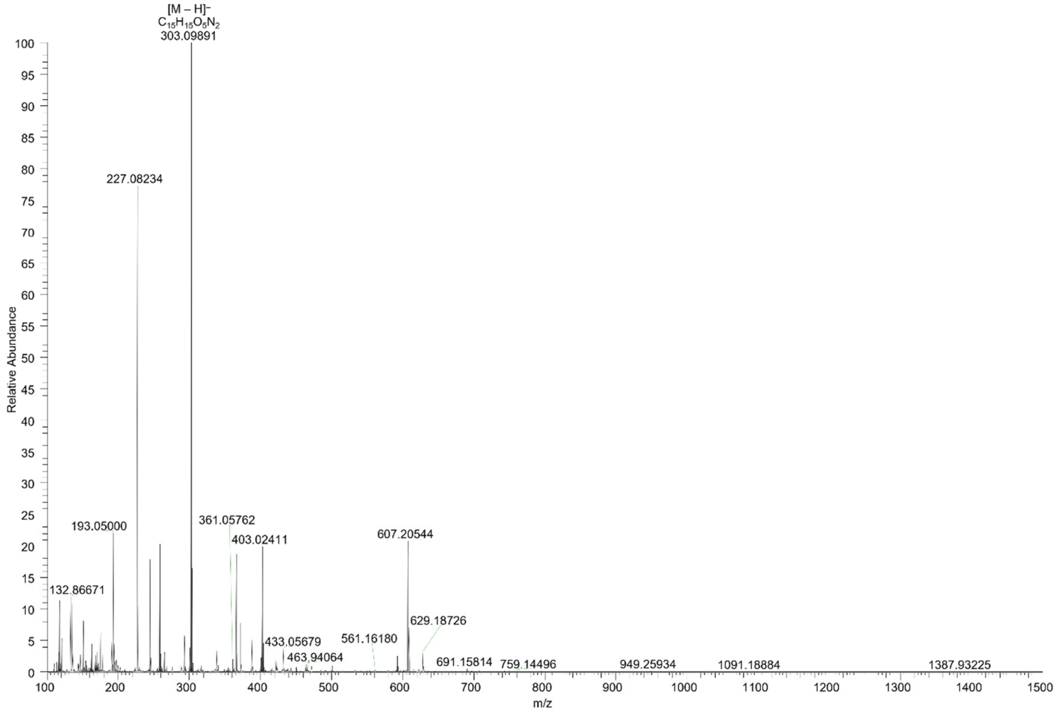

Figure S6. ESI-Q-Orbitrap-MS spectrum of **1**

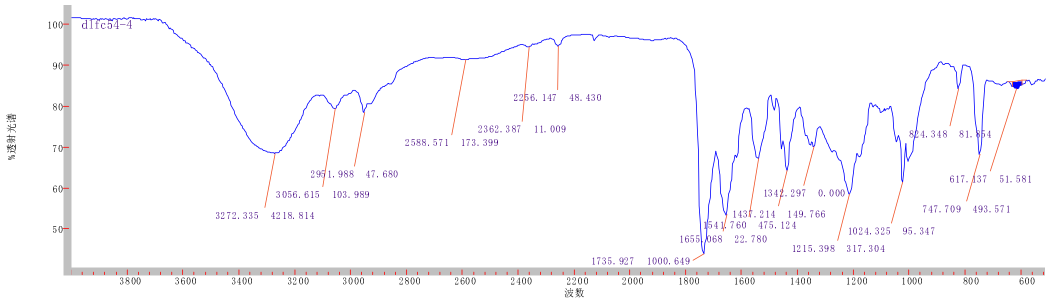

Figure S7. IR spectrum of **1**

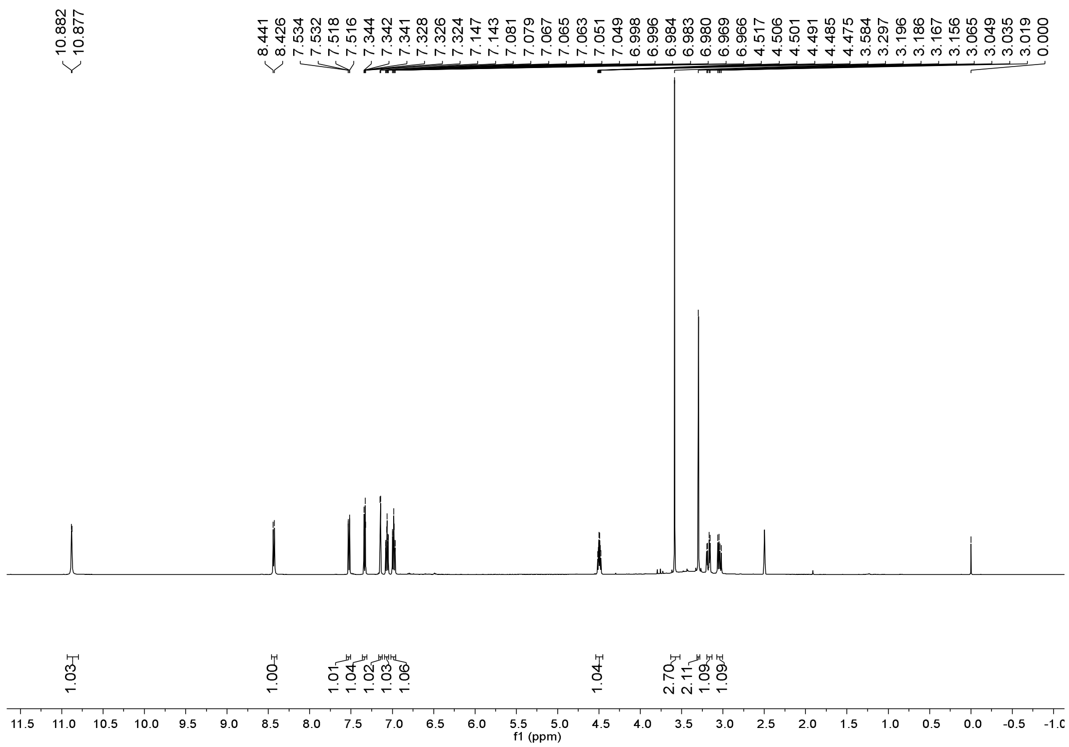

Figure S8. ^{1}H NMR (DMSO-d_6, 500 MHz) spectrum of **2**

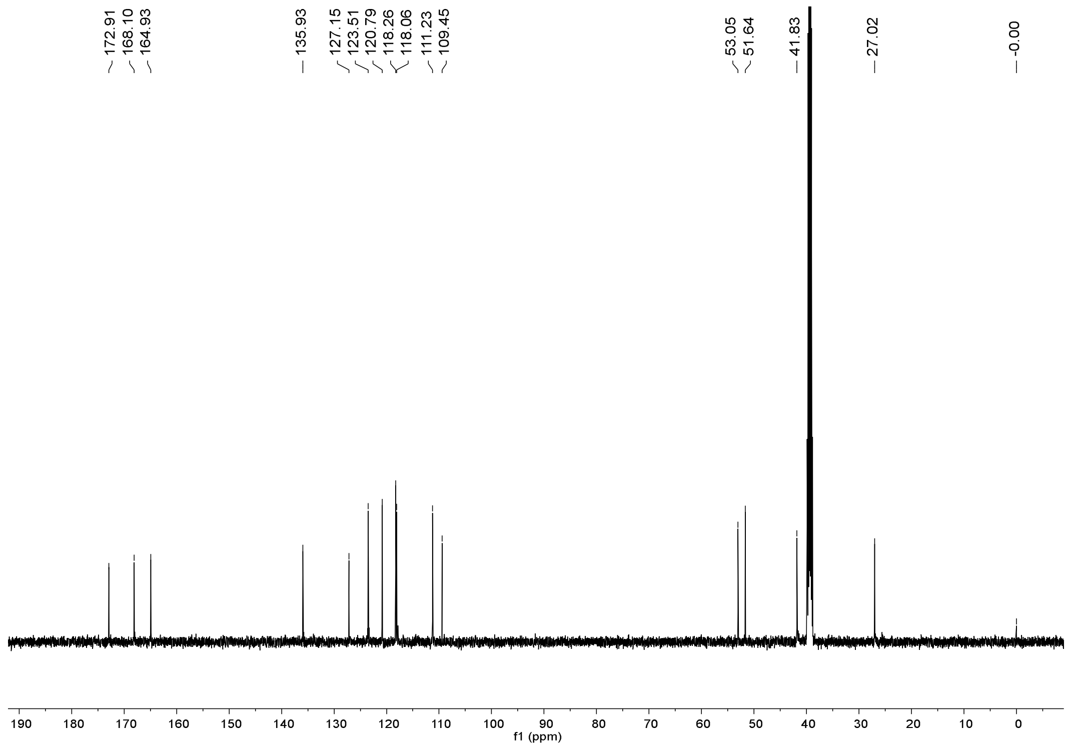

Figure S9. ^{13}C NMR (DMSO-d_6, 125 MHz) spectrum of **2**

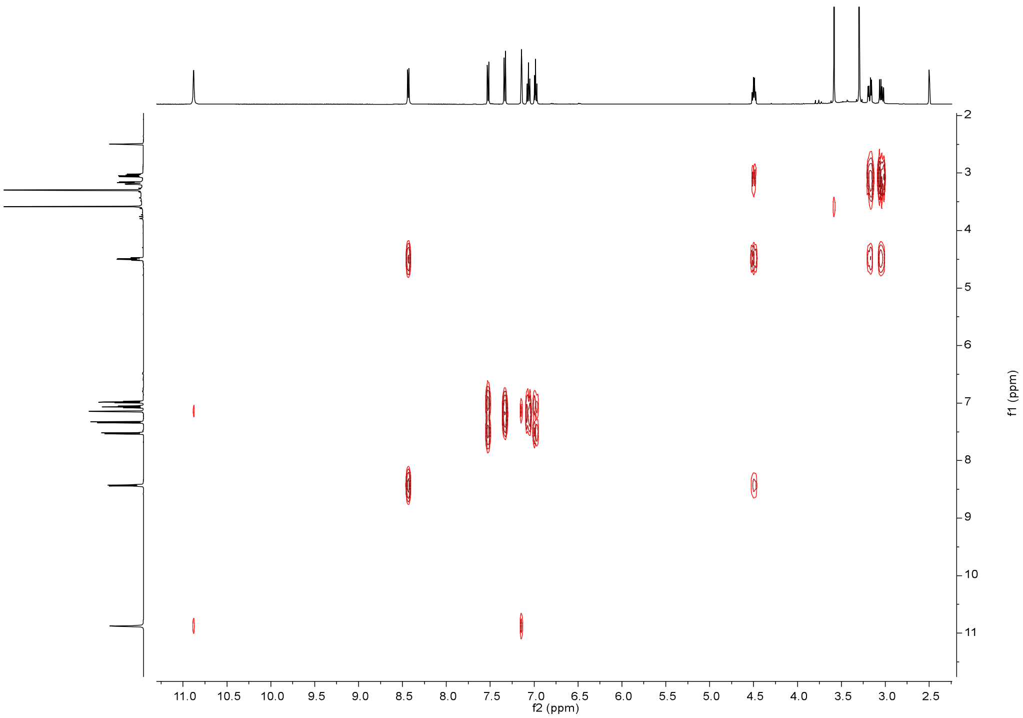

Figure S10. ^1H ^1H COSY spectrum of **2**

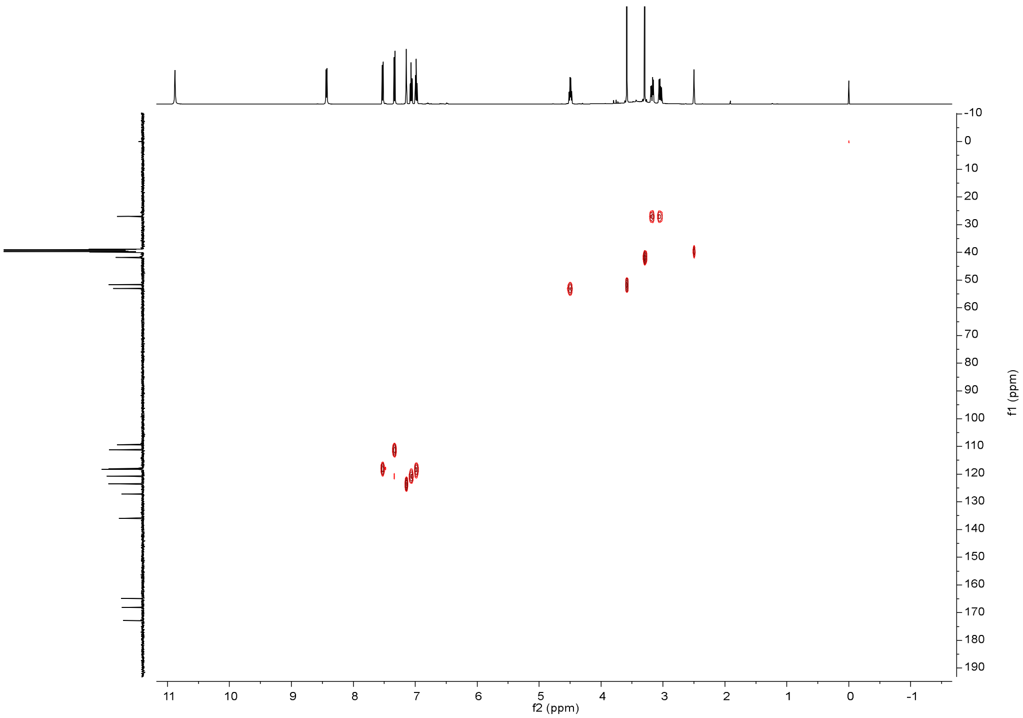

Figure S11. HSQC spectrum of **2**

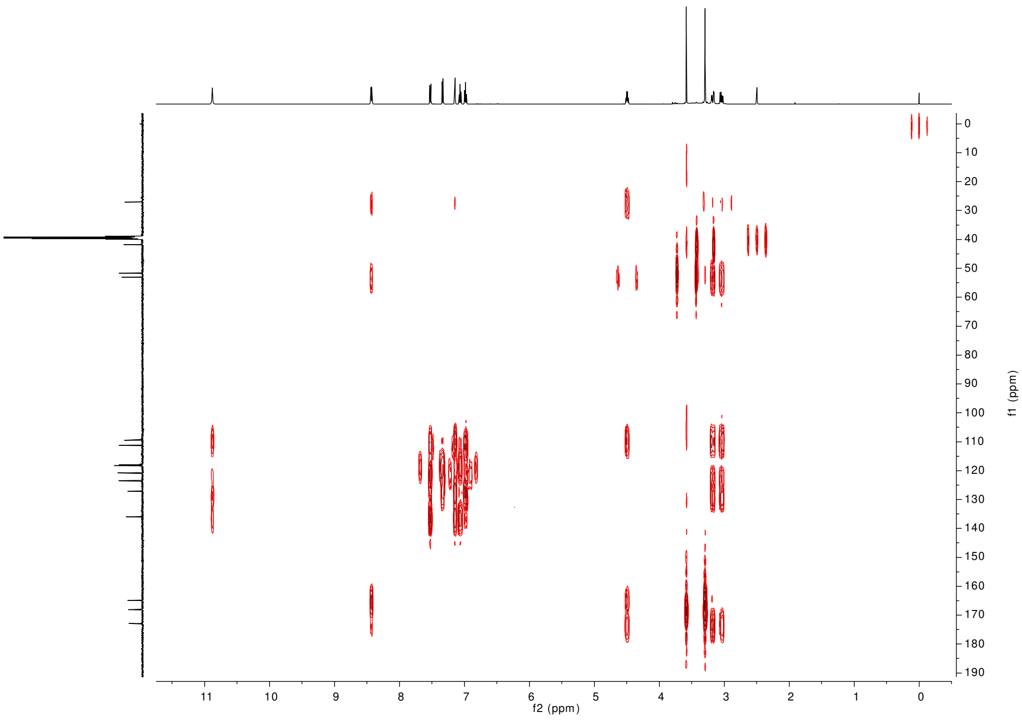

Figure S12. HMBC spectrum of **2**

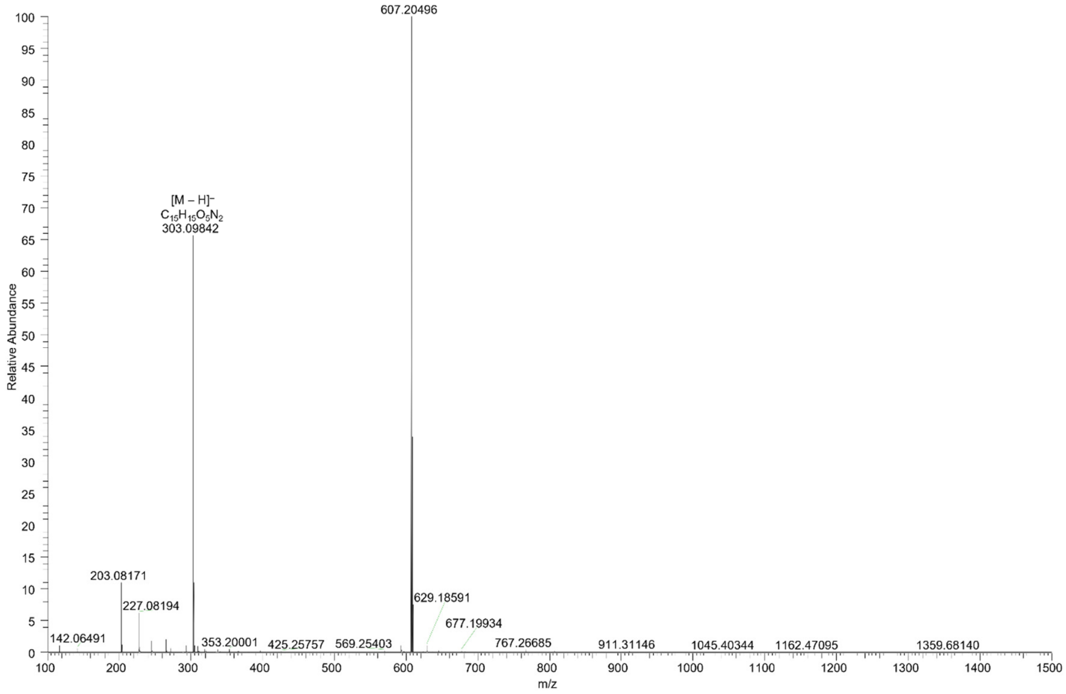

Figure S13. ESI-Q-Orbitrap-MS spectrum of **2**

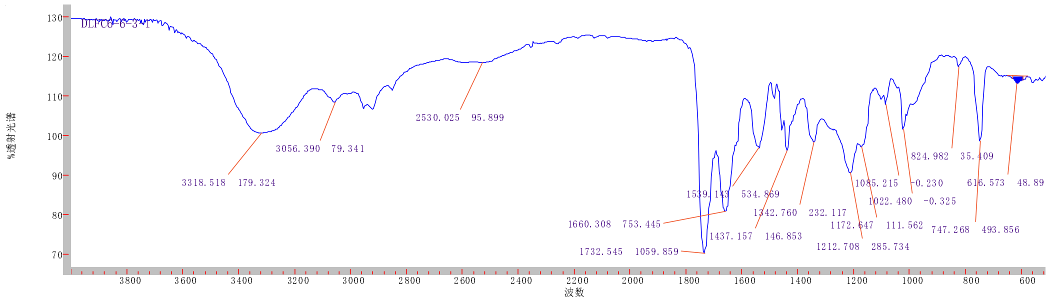

Figure S14. IR spectrum of **2**

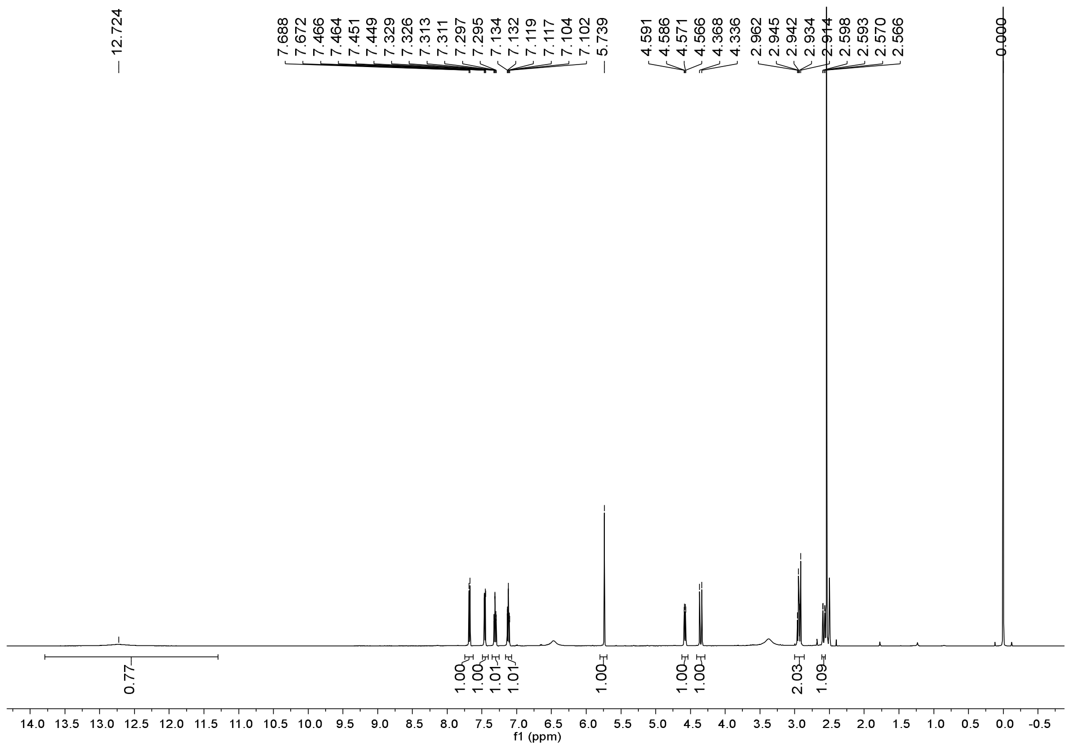

Figure S15. ^{1}H NMR (DMSO-*d*6, 500 MHz) spectrum of **3**

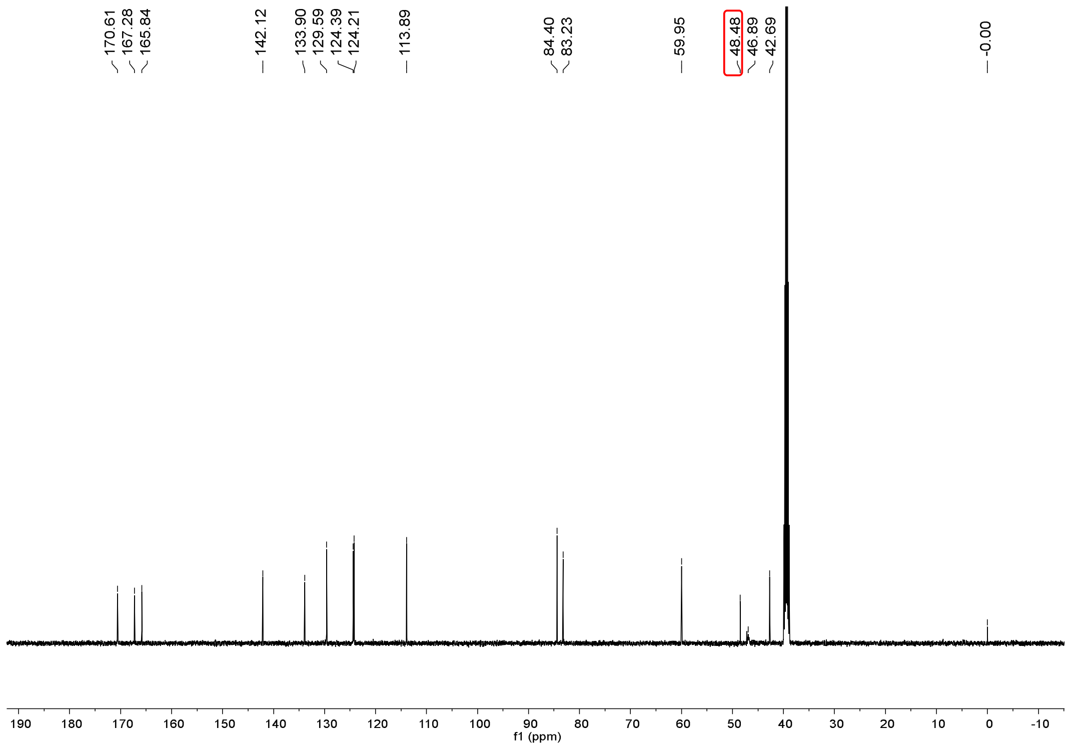

Figure S16. ^{13}C NMR (DMSO-*d*6, 125 MHz) spectrum of **3**

Note: δ 48.5 is the signal of residual methanol

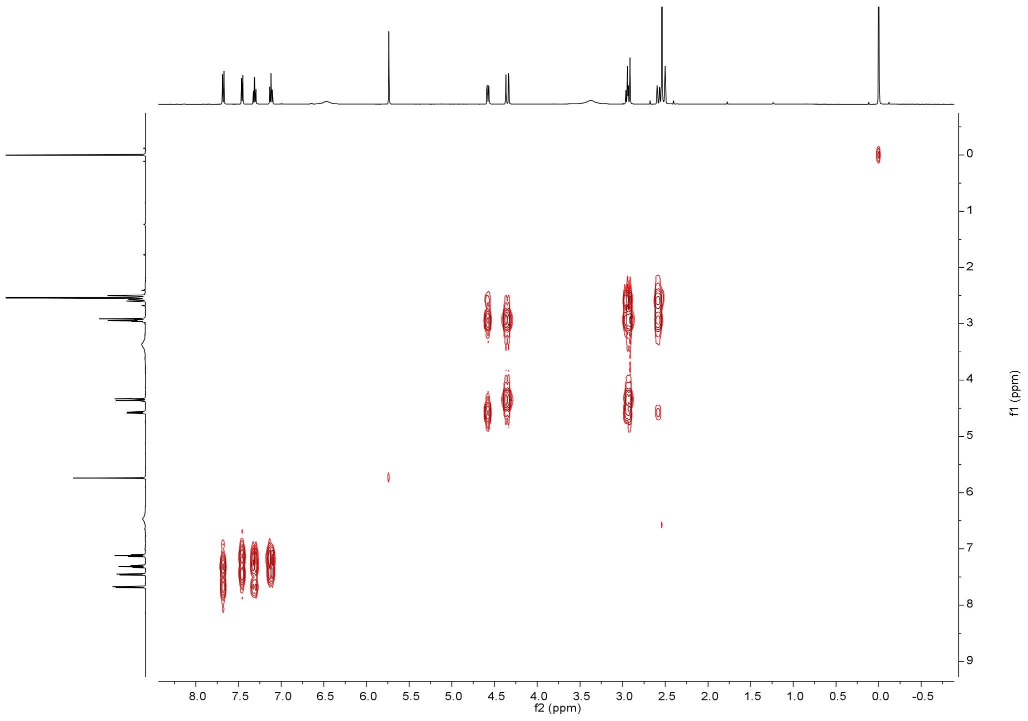

Figure S17. ^{1}H ^{1}H COSY spectrum of **3**

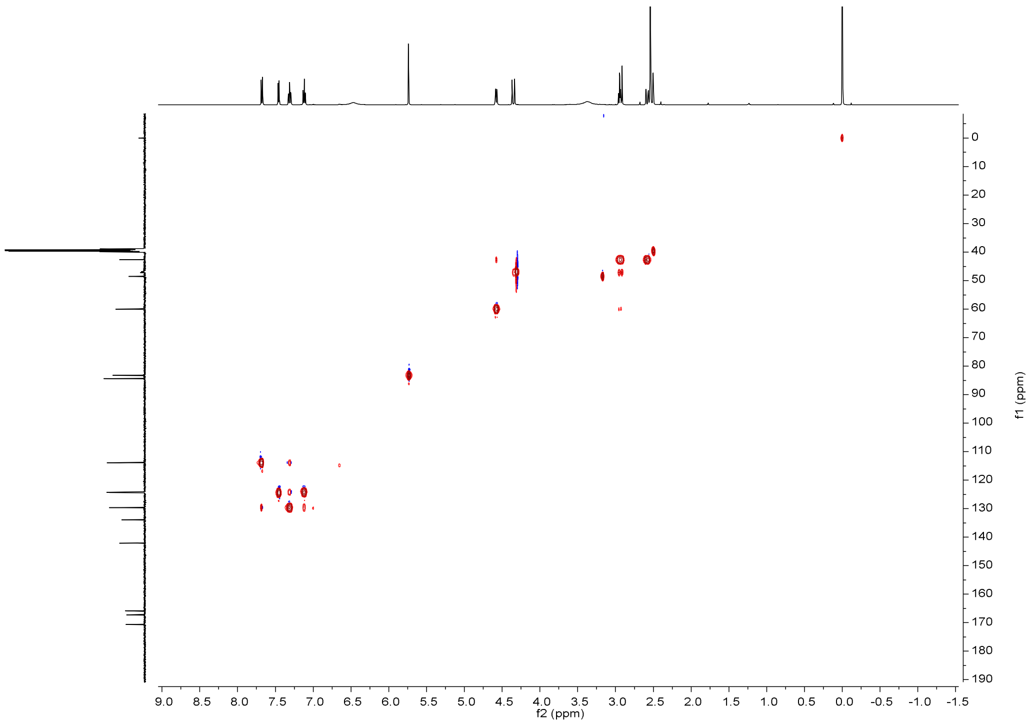

Figure S18. HSQC spectrum of **3**

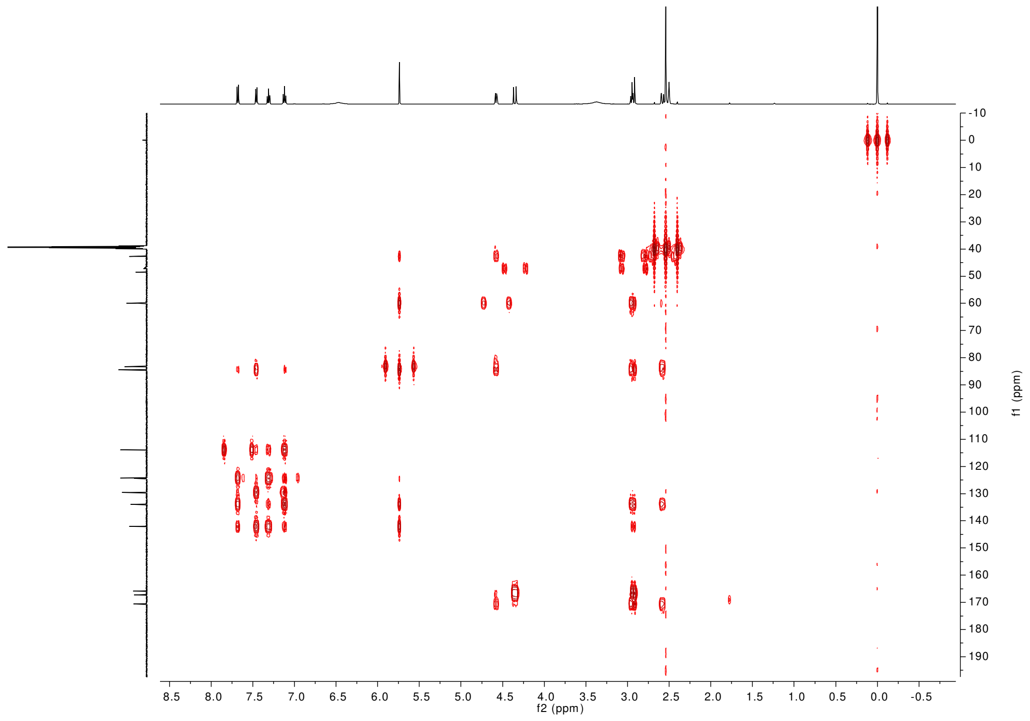

Figure S19. HMBC spectrum of **3**

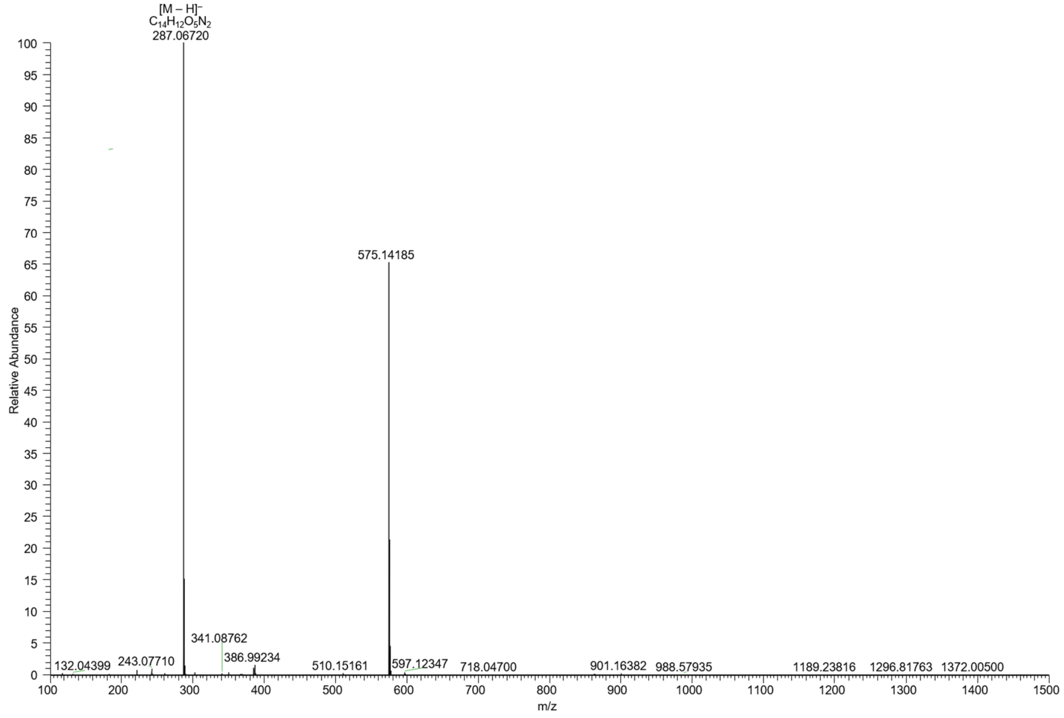

Figure S20. ESI-Q-Orbitrap-MS spectrum of **3**

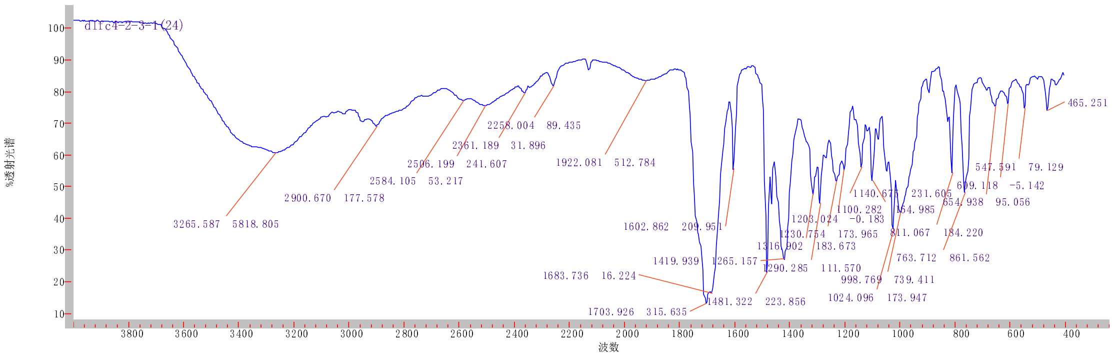

Figure S21. IR spectrum of **3**

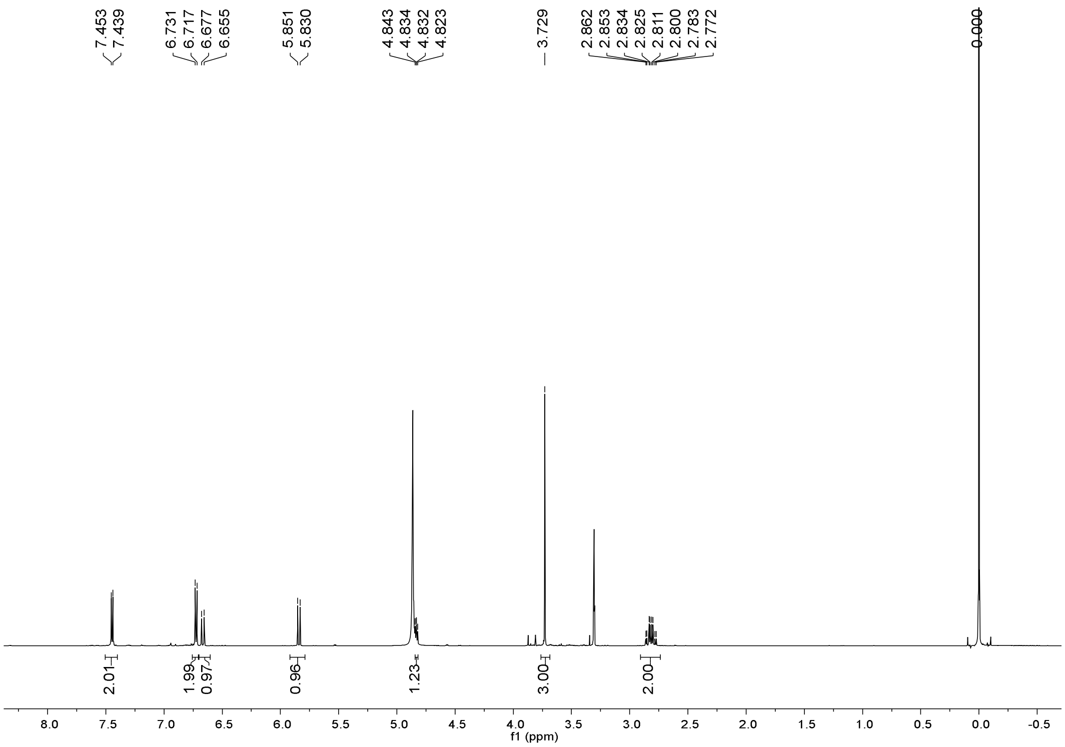

Figure S22 ^{1}H NMR (CD_3OD, 600 MHz) spectrum of **4**

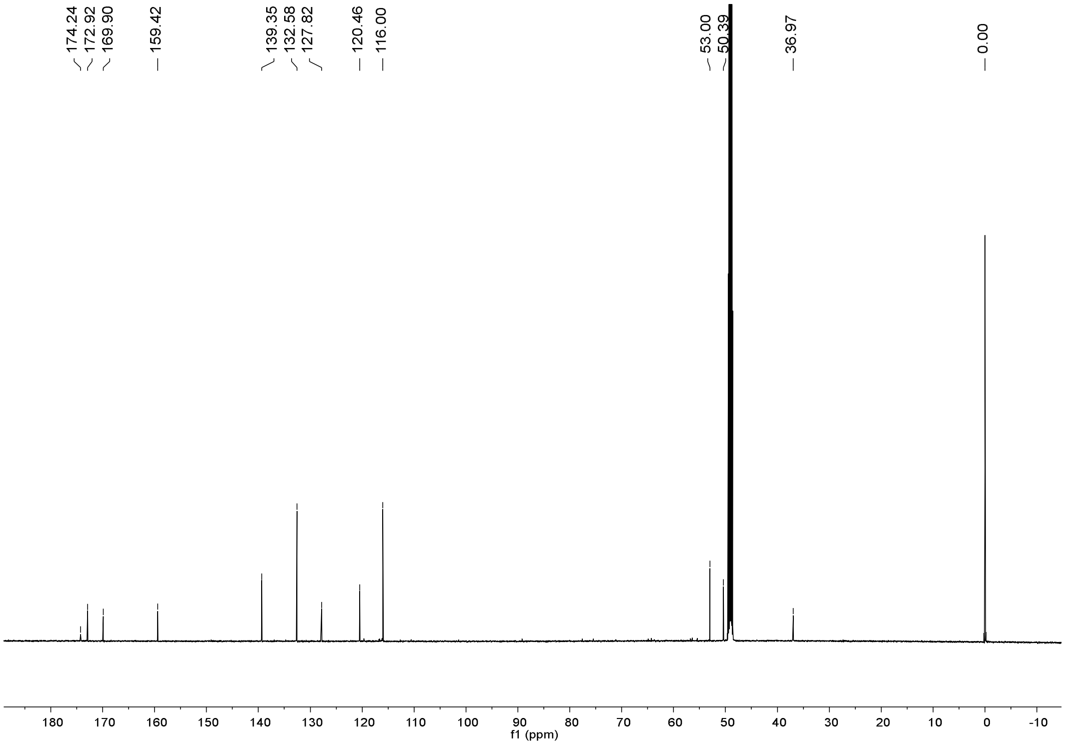

Figure S23 ^{13}C NMR (CD_3OD, 150 MHz) spectrum of **4**

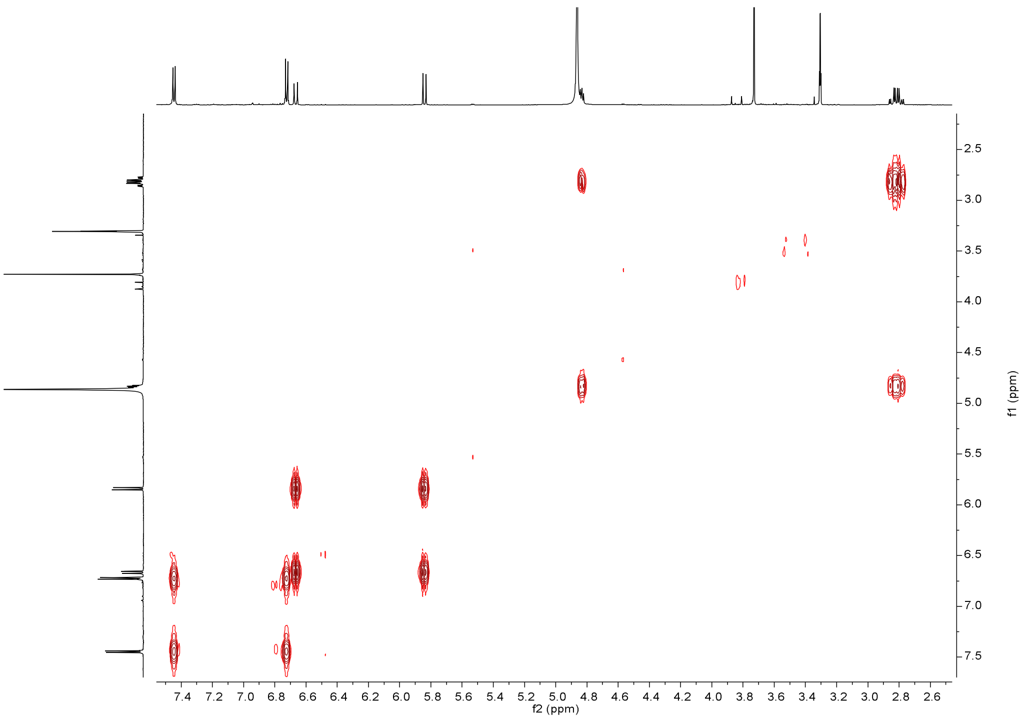

Figure S24 ^{1}H ^{1}H COSY spectrum of **4**

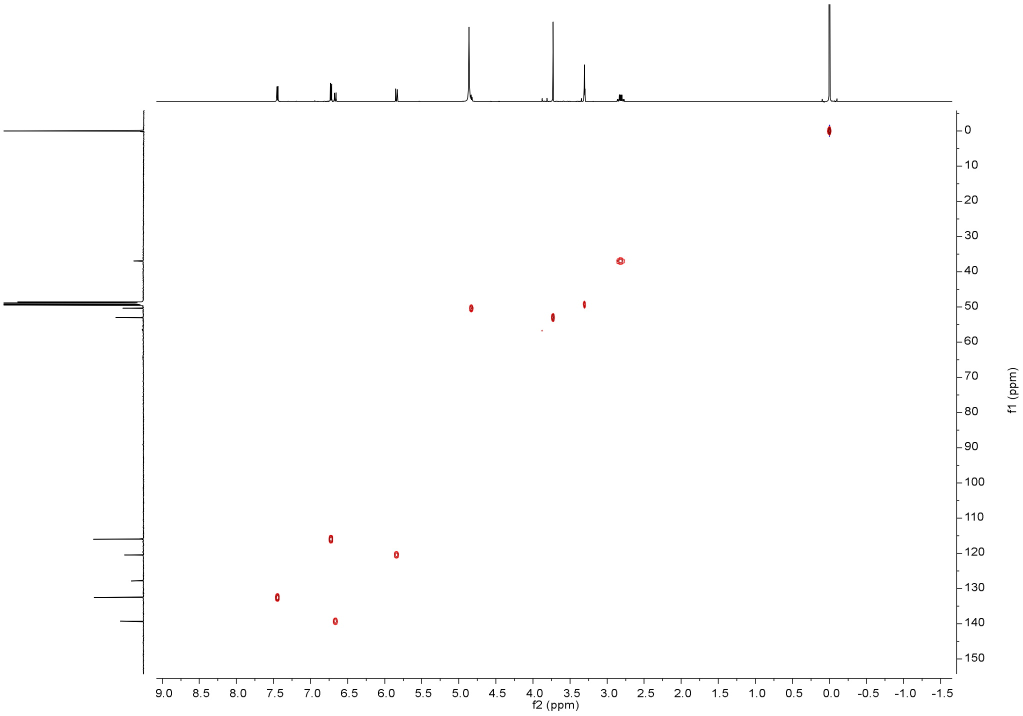

Figure S25 HSQC spectrum of **4**

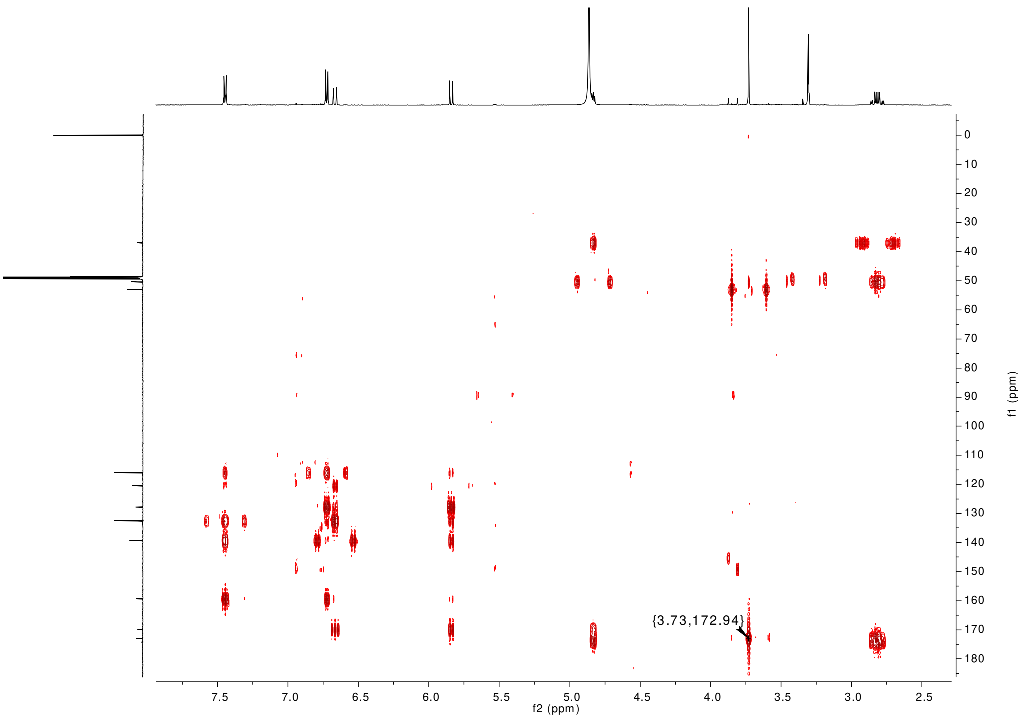

Figure S26 HMBC spectrum of **4**

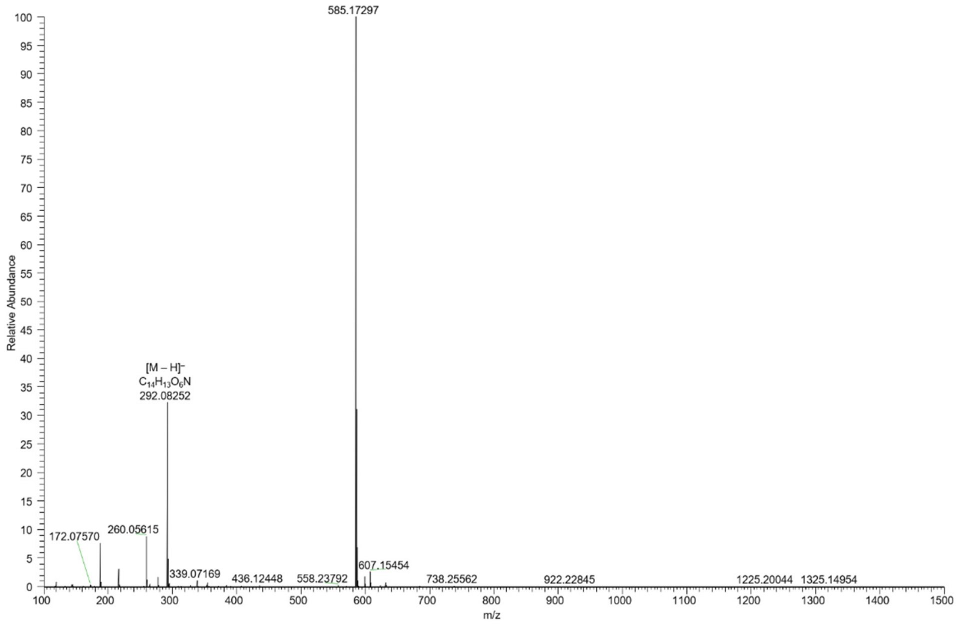

Figure S27 ESI-Q-Orbitrap-MS spectrum of **4**

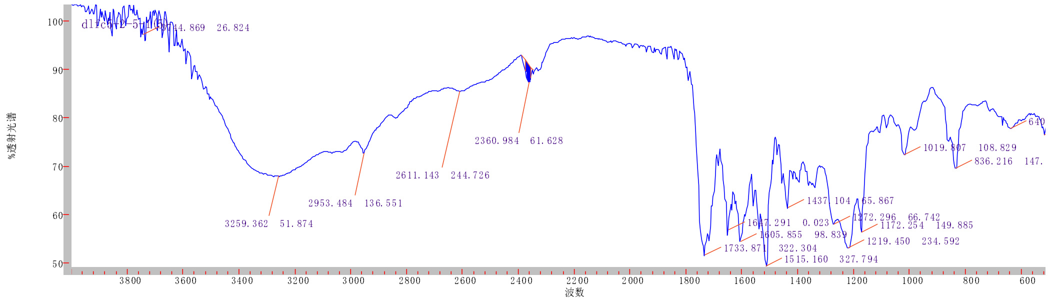

Figure S28 IR spectrum of **4**

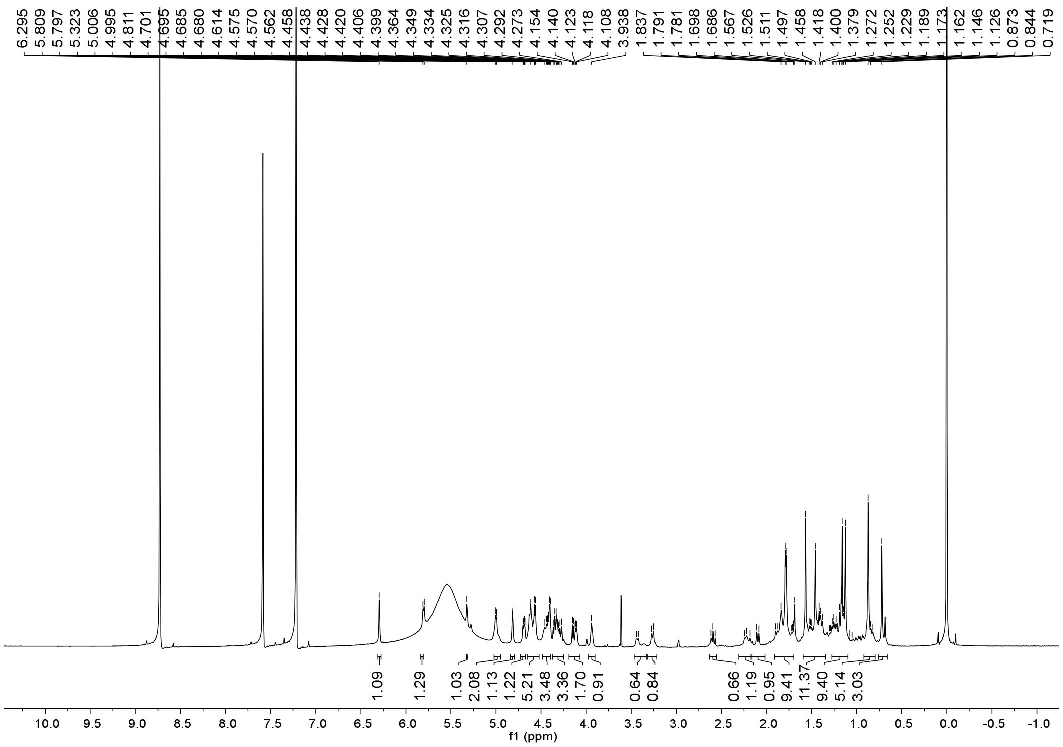

Figure S29 ^{1}H NMR (C_5D_5N, 600 MHz) spectrum of **5**

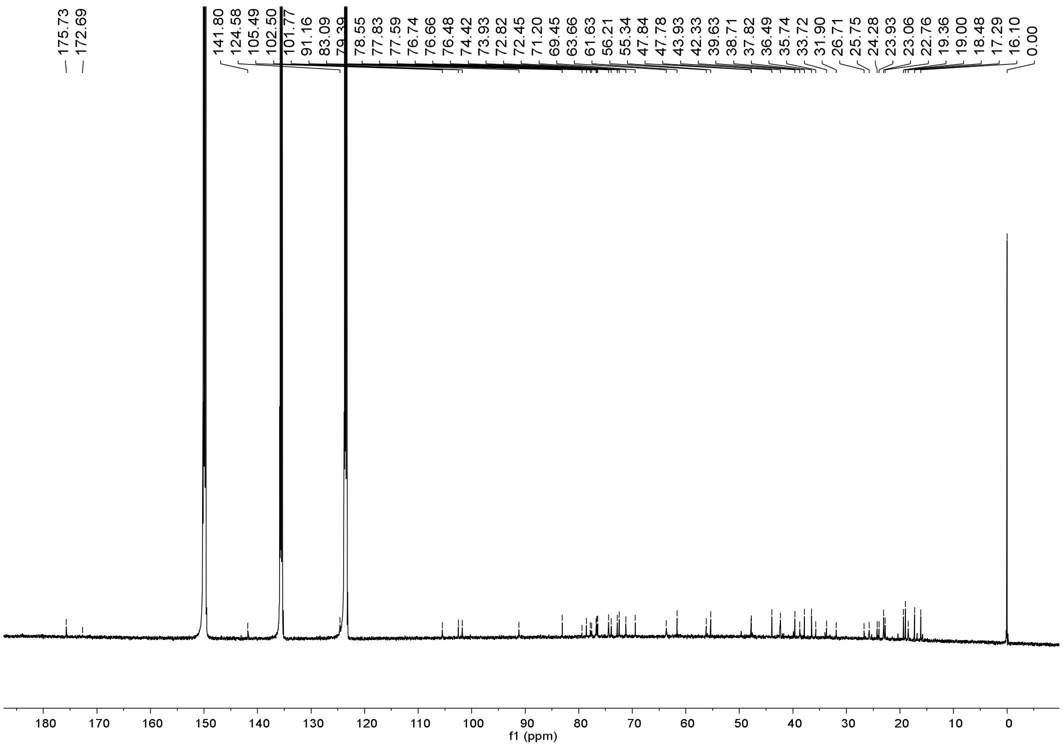

Figure S30 ^{13}C NMR (C_5D_5N, 150 MHz) spectrum of **5**

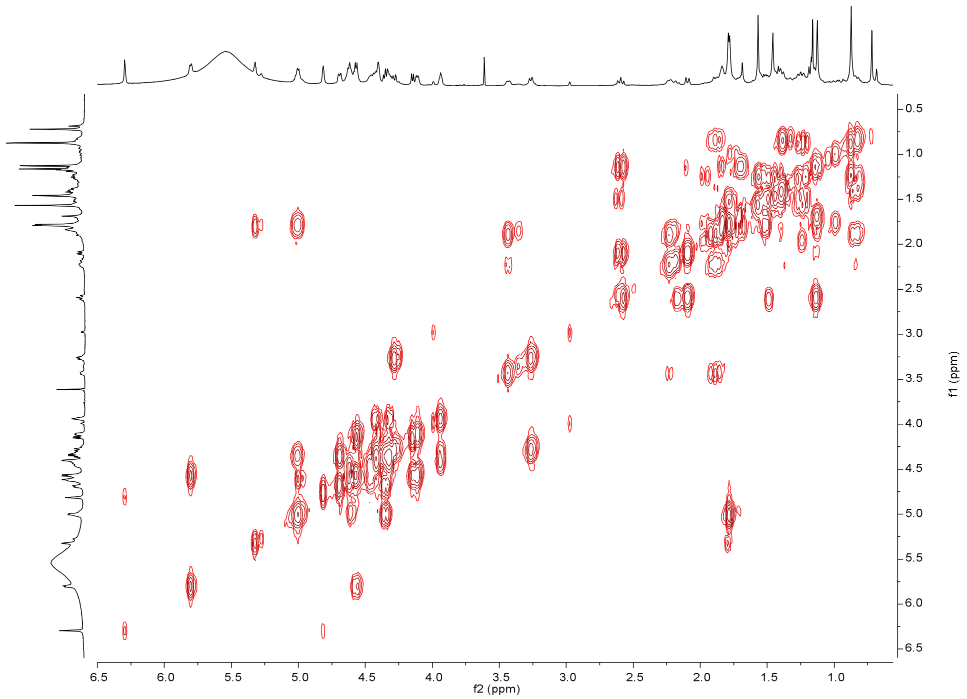

Figure S31 ^{1}H ^{1}H COSY spectrum of **5**

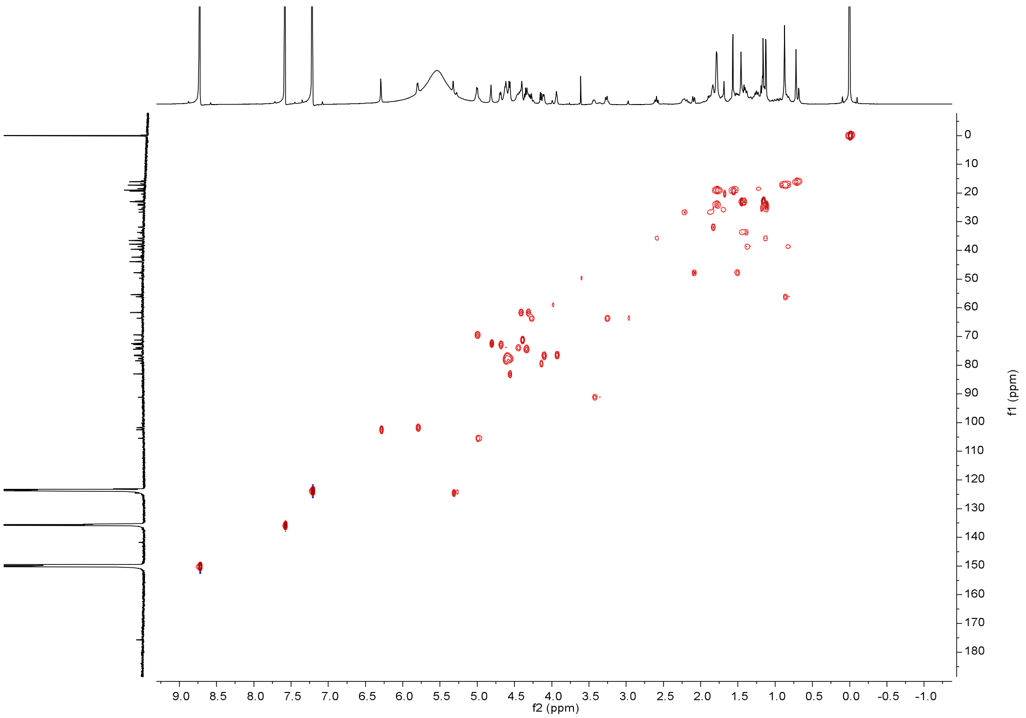

Figure S32 HSQC spectrum of **5**

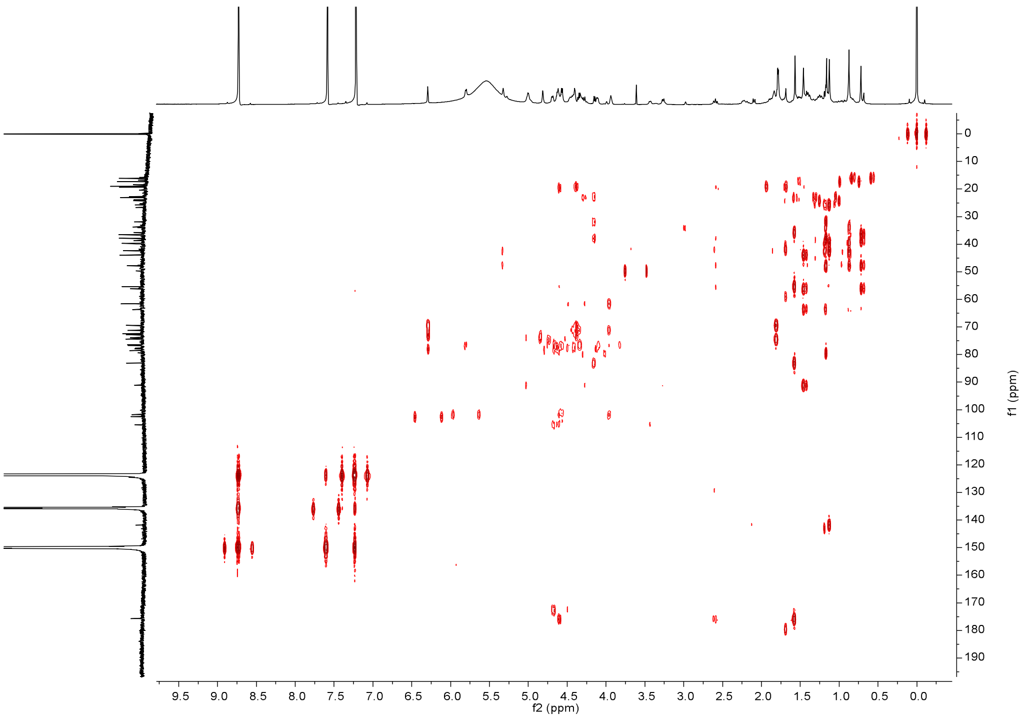

Figure S33 HMBC spectrum of **5**

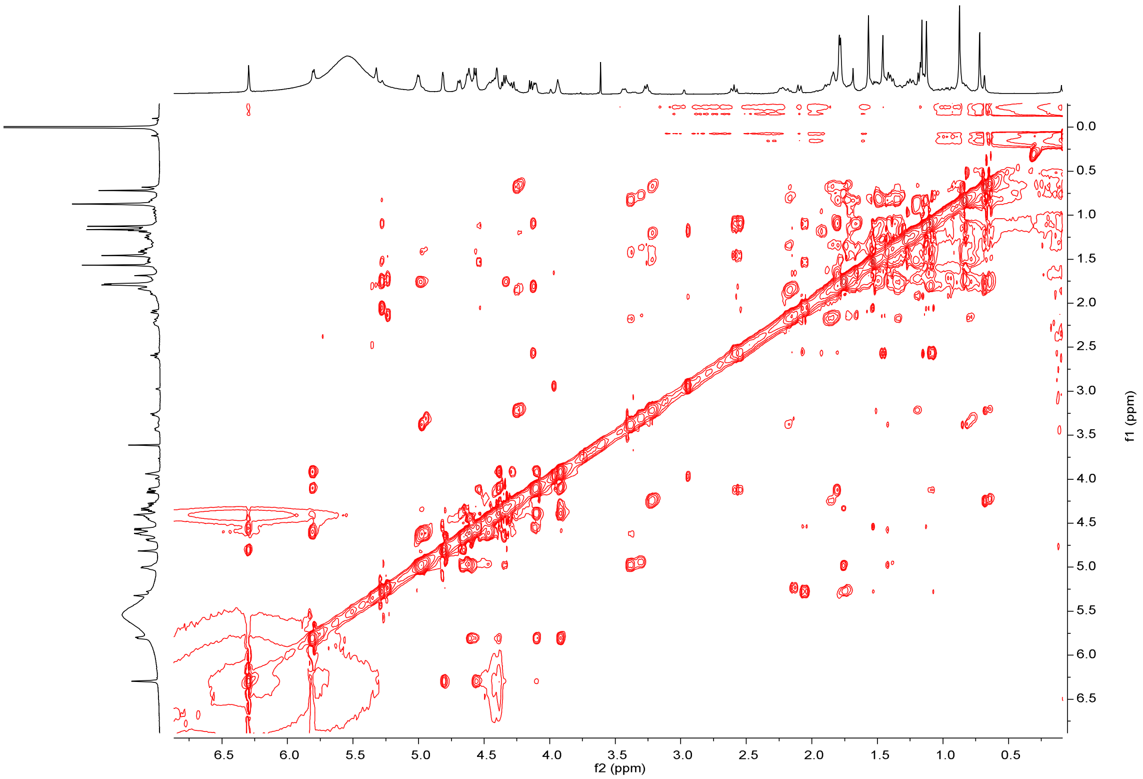

Figure S34 NOESY spectrum of **5**

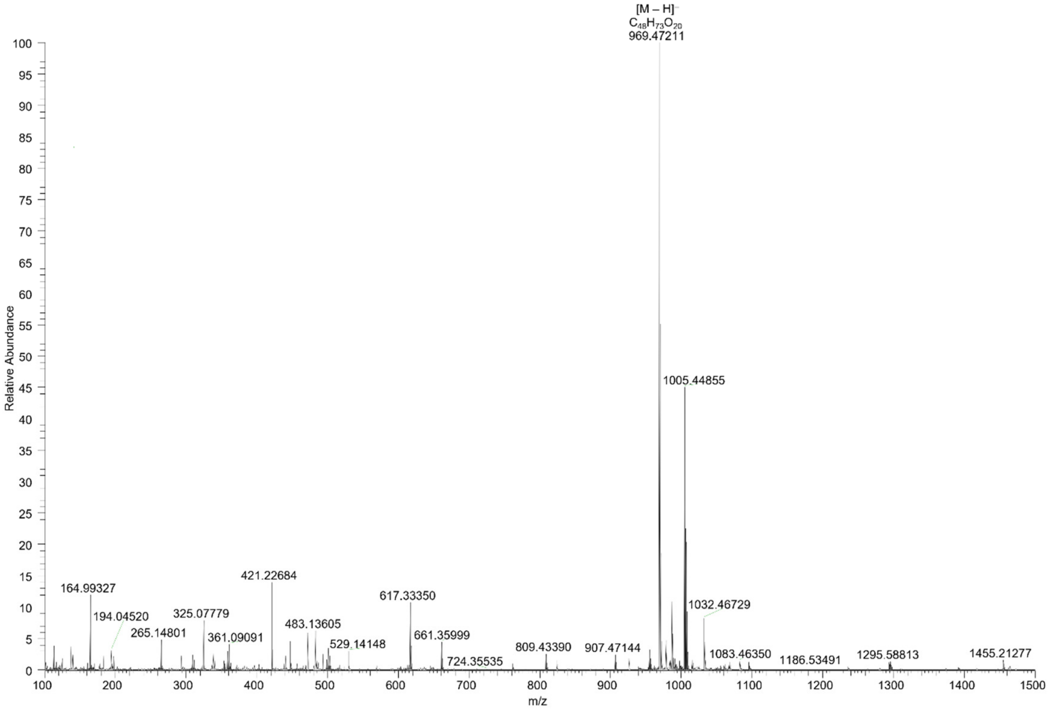

Figure S35 ESI-Q-Orbitrap-MS spectrum of **5**

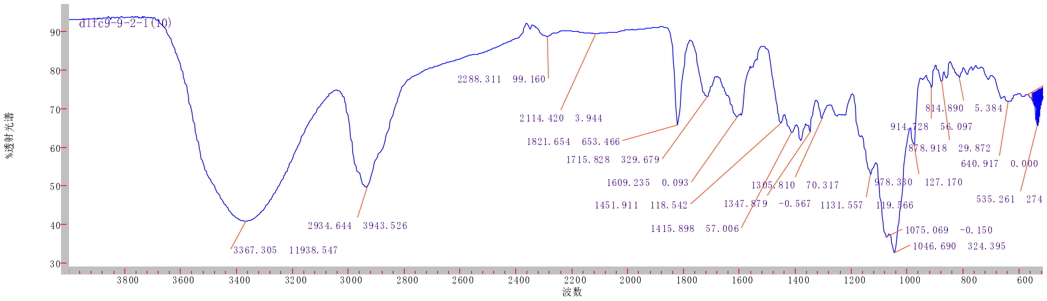

Figure S36 IR spectrum of **5**

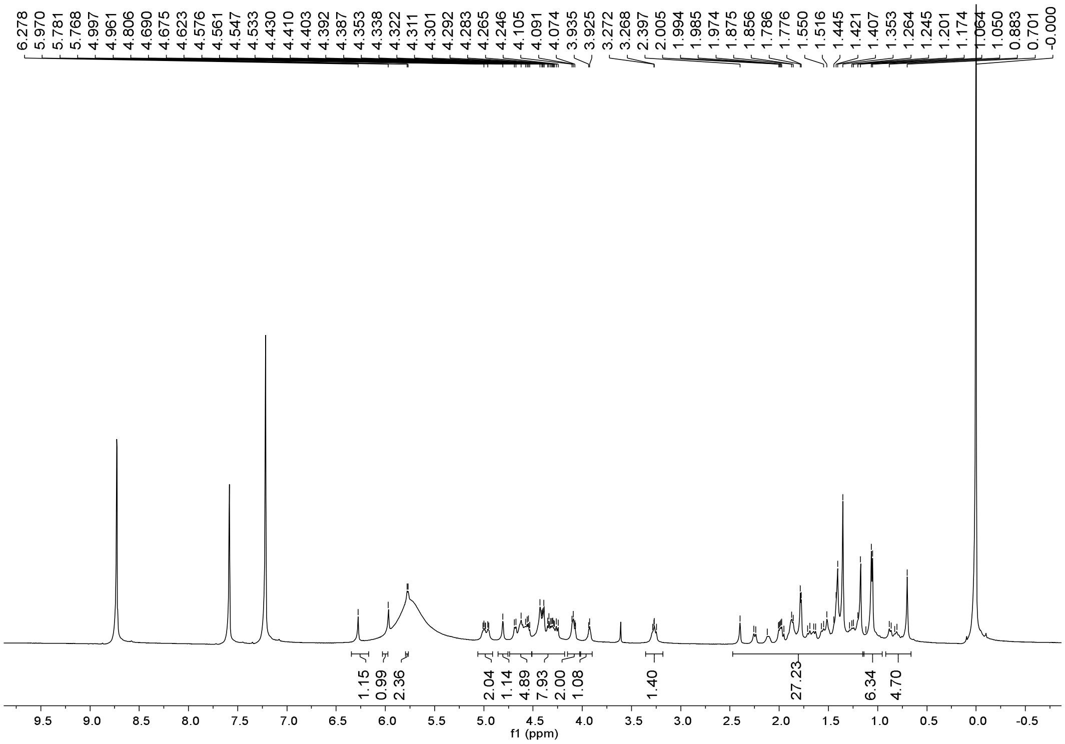

Figure S37 ^{1}H NMR (C_5D_5N, 600 MHz) spectrum of **6**

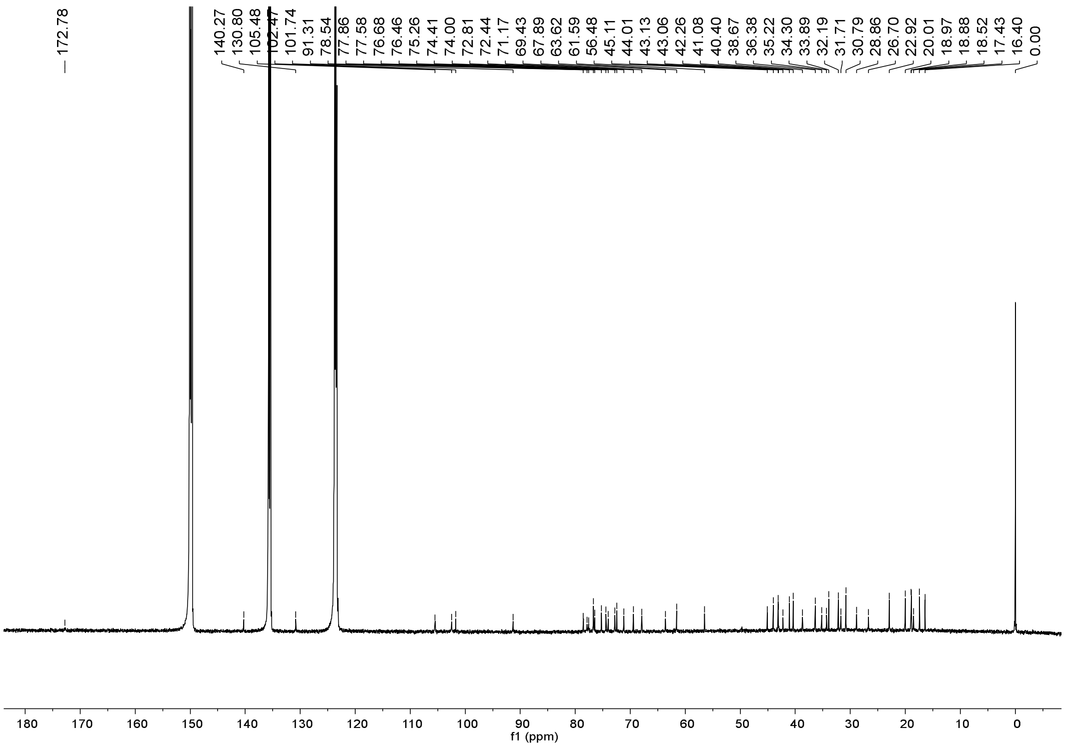

Figure S38 ^{13}C NMR (C_5D_5N, 150 MHz) spectrum of **6**

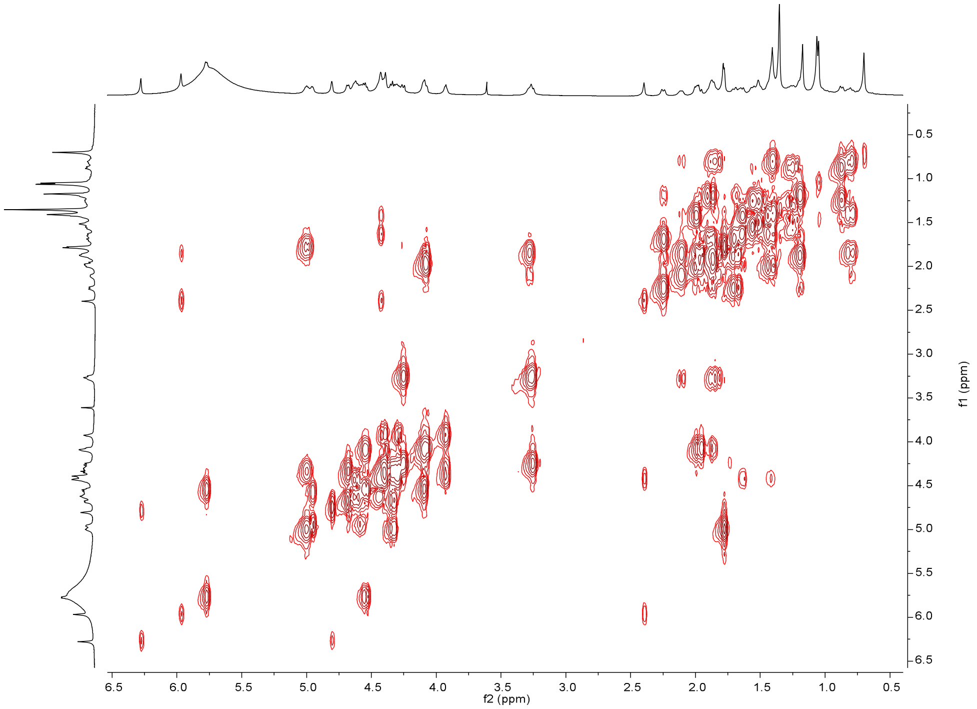

Figure S39 ^{1}H ^{1}H COSY spectrum of **6**

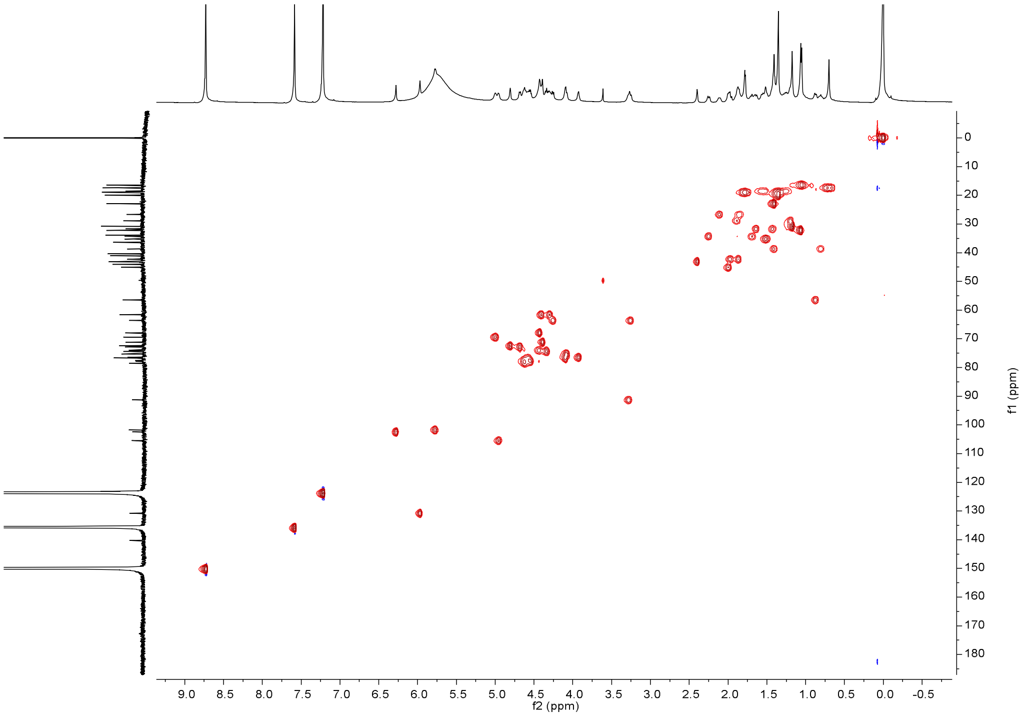

Figure S40 HSQC spectrum of **6**

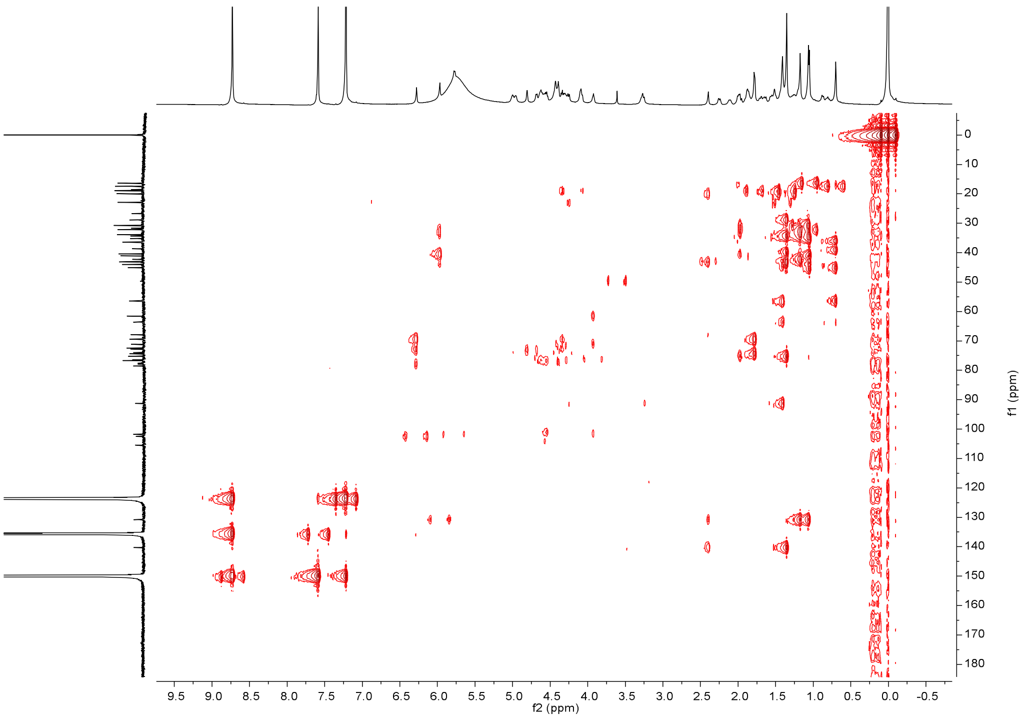

Figure S41 HMBC spectrum of **6**

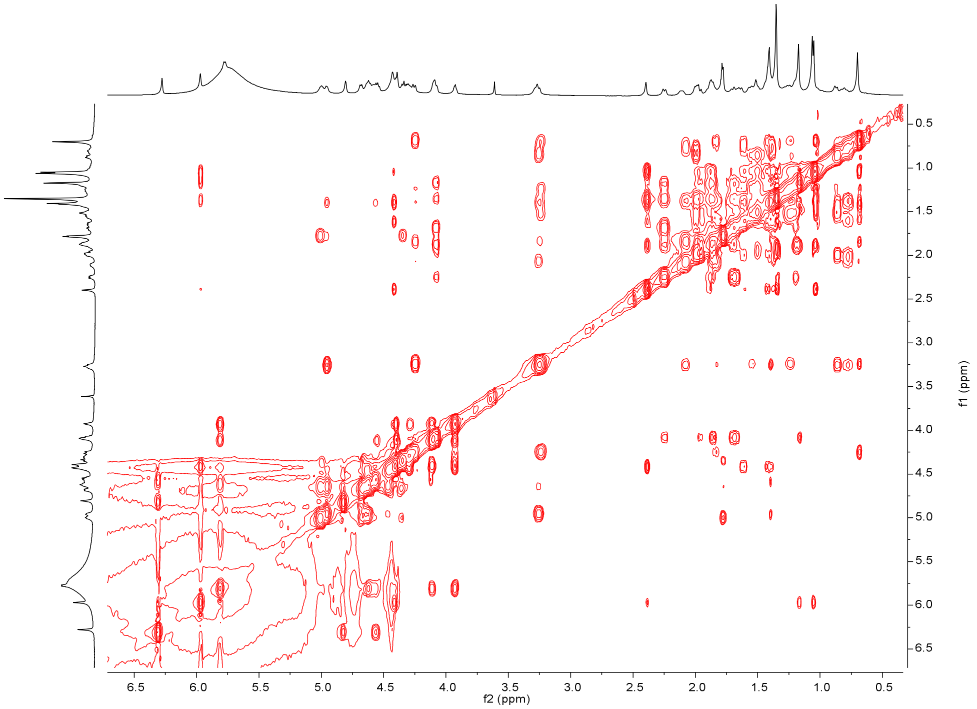

Figure S42 NOESY spectrum of **6**

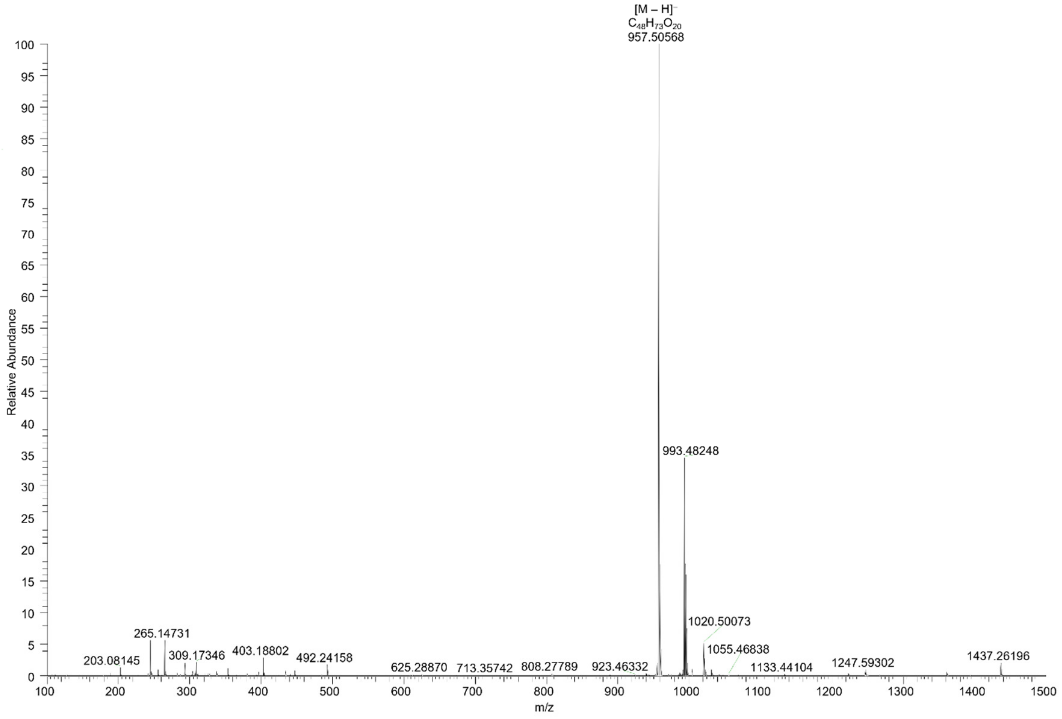

Figure S43 ESI-Q-Orbitrap-MS spectrum of **6**

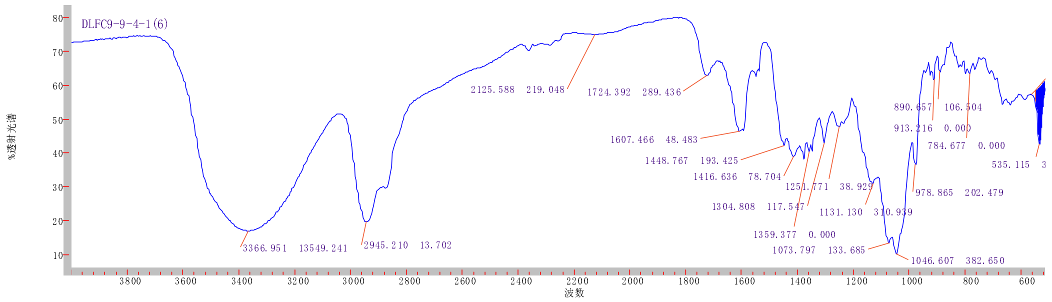

Figure S44 IR spectrum of **6**

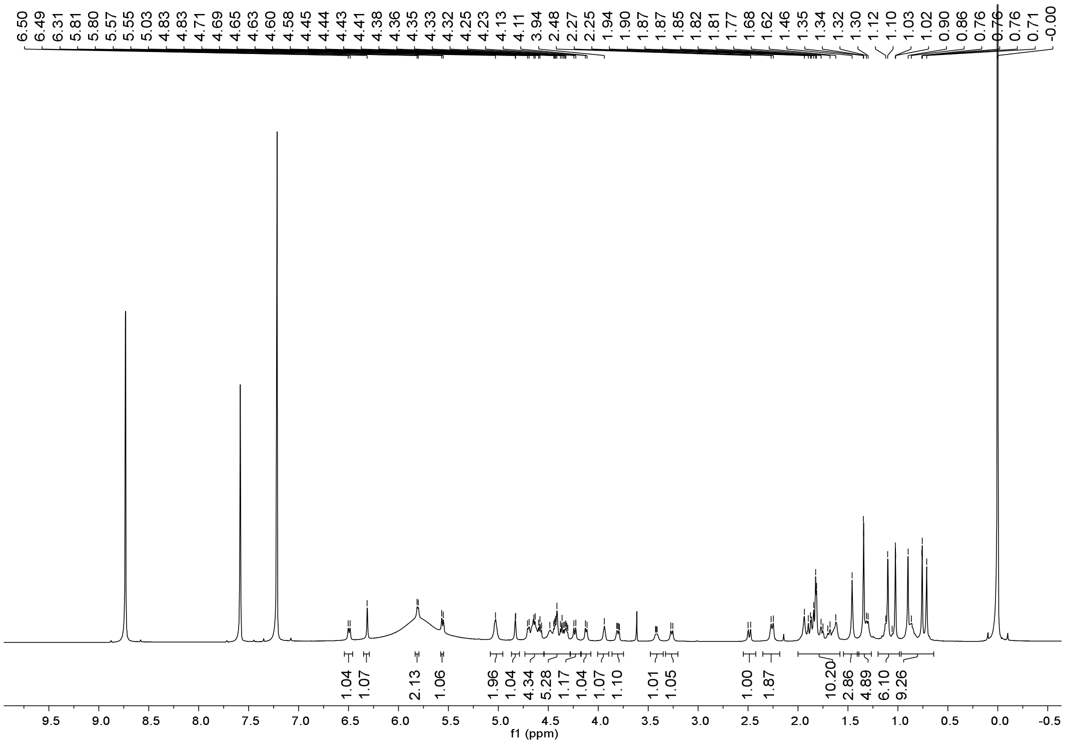

Figure S45 ^1H NMR (C_5D_5N, 600 MHz) spectrum of **7**

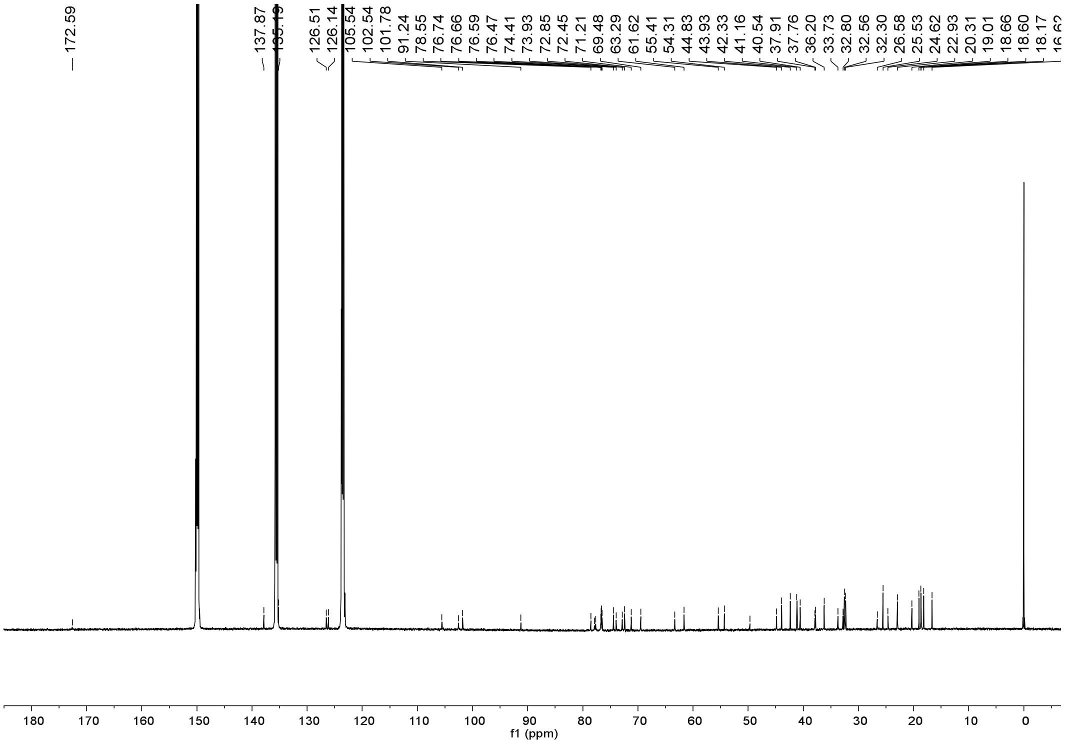

Figure S46 ^{13}C NMR (C_5D_5N, 150 MHz) spectrum of **7**

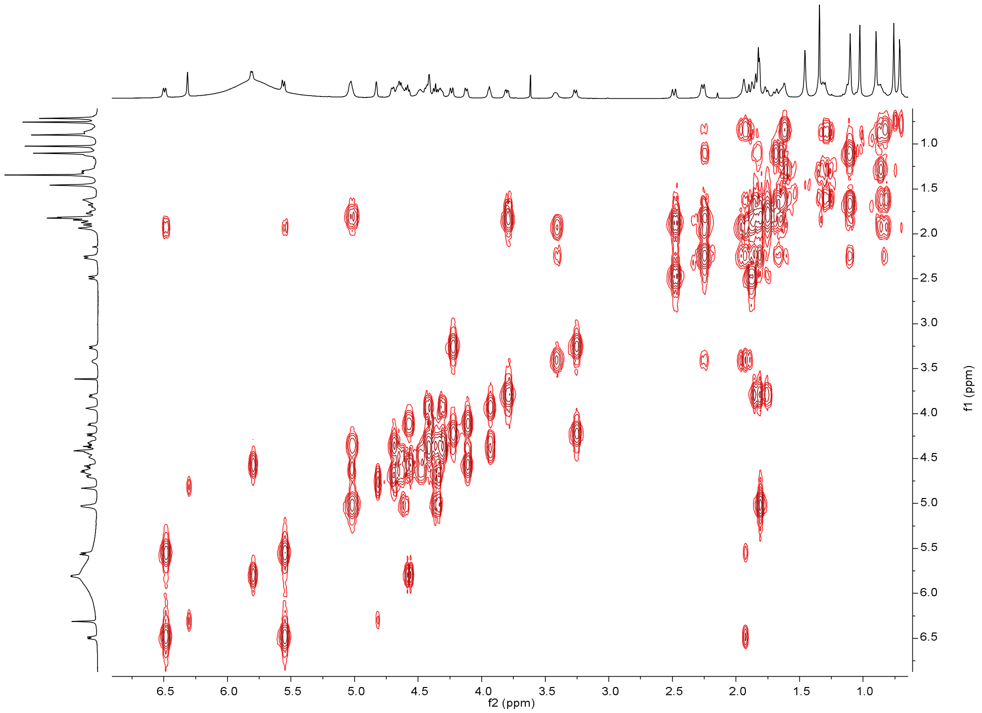

Figure S47 ^{1}H ^{1}H COSY spectrum of **7**

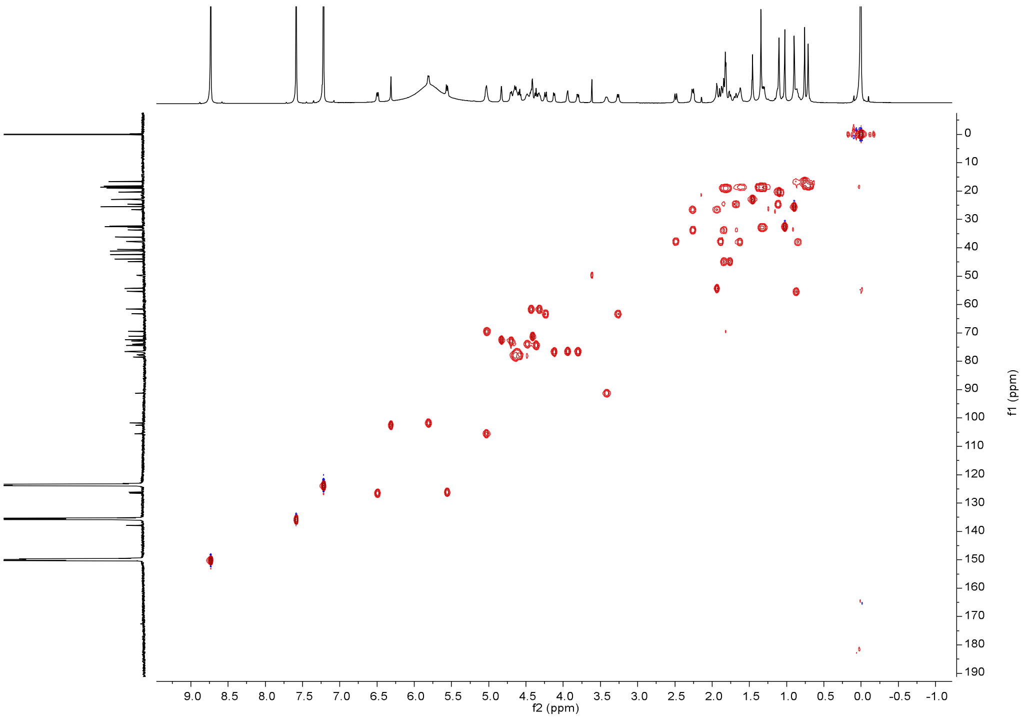

Figure S48 HSQC spectrum of **7**

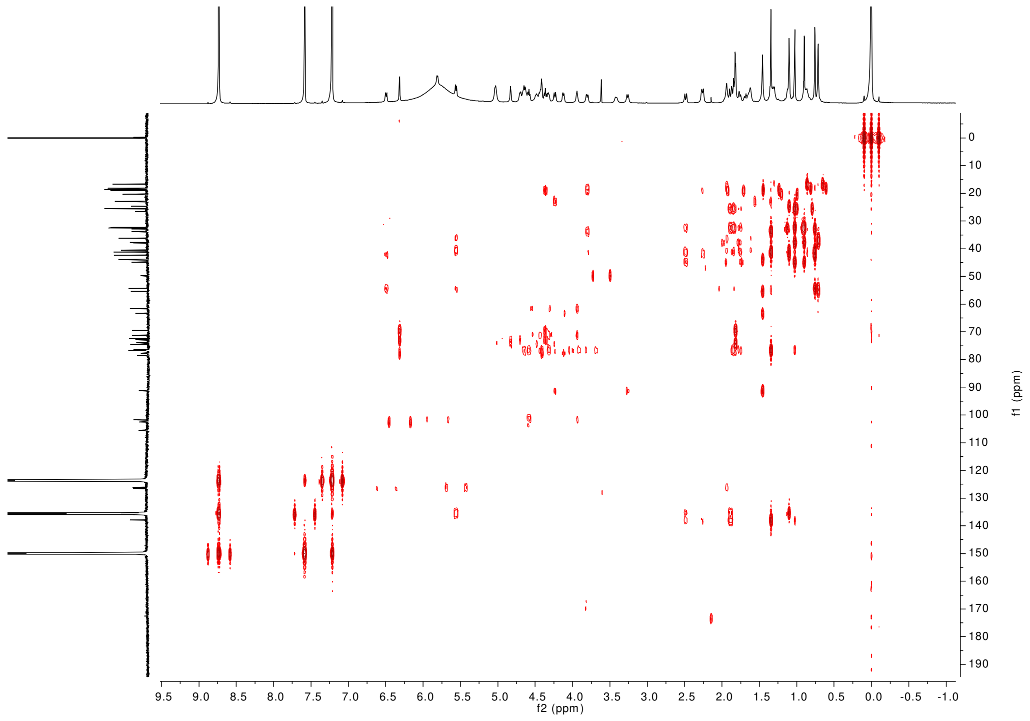

Figure S49 HMBC spectrum of **7**

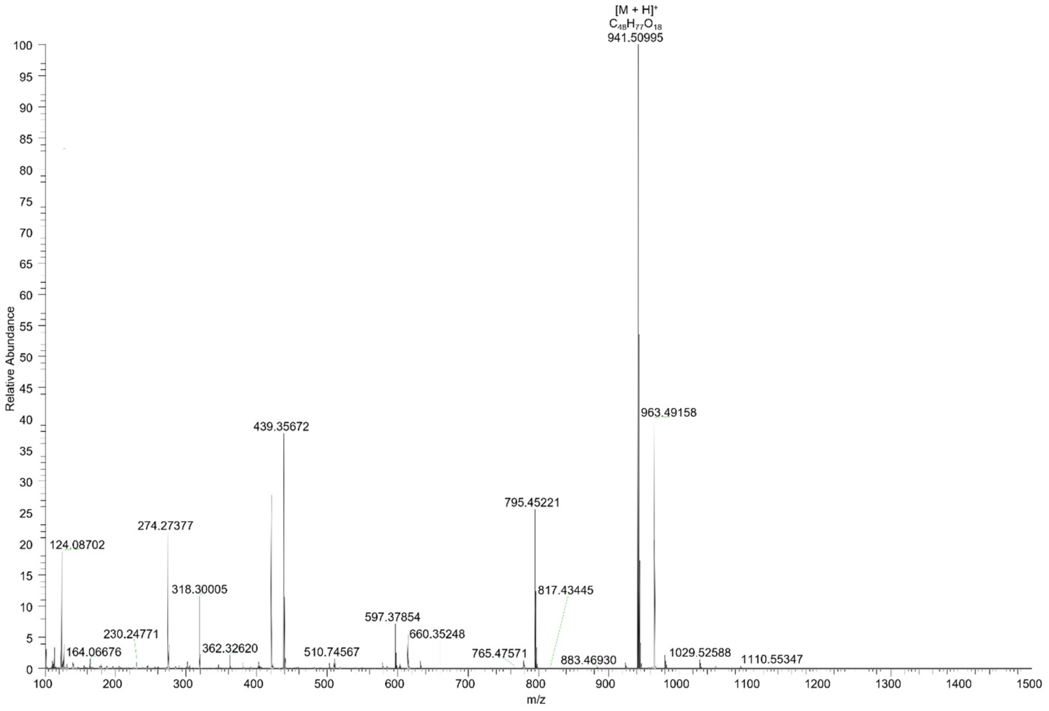

Figure S50 ESI-Q-Orbitrap-MS spectrum of **7**

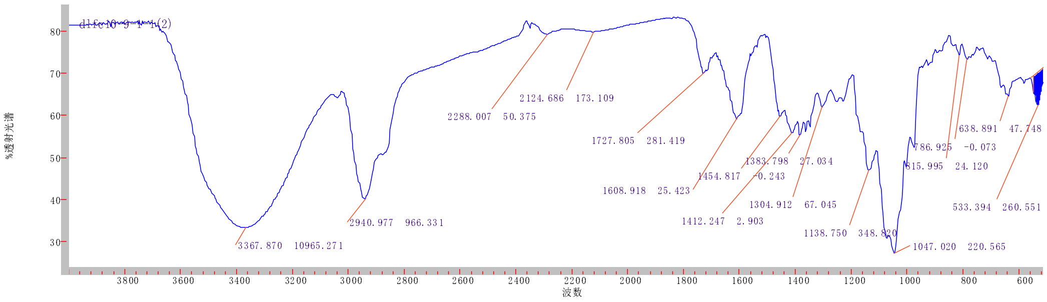

Figure S51 IR spectrum of **7**

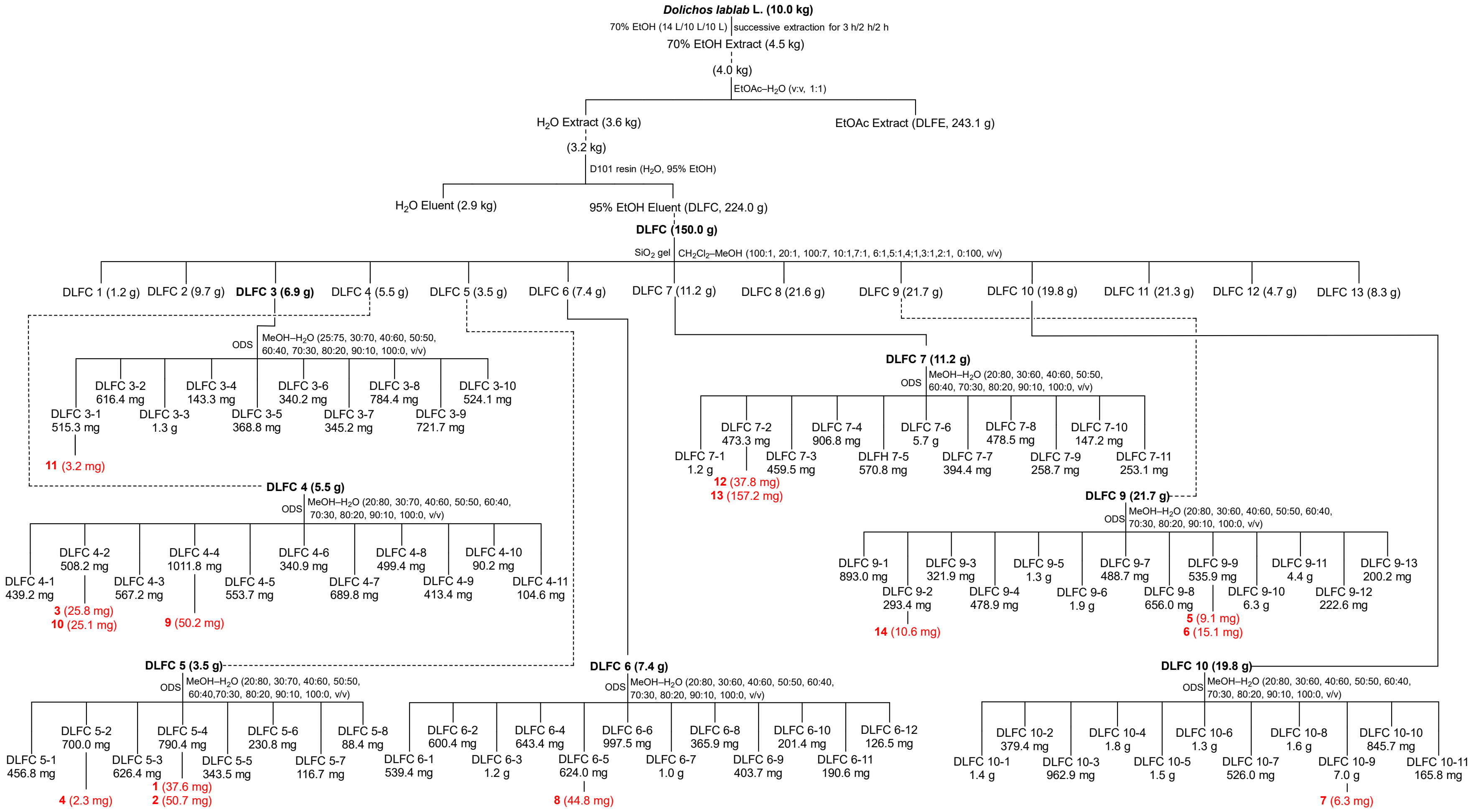

Figure S52. Schematic representation of extraction and separation of compounds **1**–**14**.

The physical data of compounds 8–14

N-Malonyl-L-tryptophan (**8**)

White powder; $[\alpha]_D^{25}$ +14.9 (*cocn* 0.23, MeOH); CD (*conc* 0.001 M, MeOH) mdeg (λ_{nm}) : +0.67 (282), +0.47 (222), +0.15 (261), +10.62 (222); ^{1}H NMR (CD_3OD, 500 MHz) δ_H: 7.12 (1H, s, H-2), 7.56 (1H, br. d, *ca.* *J* = 8 Hz, H-4), 7.00 (1H, t like, *ca.* *J* = 8 Hz, H-5), 7.07 (1H, t like, *ca.* *J* = 8 Hz, H-6), 7.31 (1H, br. d, *ca.* *J* = 8 Hz, H-7), [3.24 (1H, dd, *J* = 7.0, 15.0 Hz), 3.34 (1H, dd, *J* = 5.5, 15.0 Hz), H_2-10], 4.77 (1H, t like, *ca.* *J* = 6 Hz, H-11), 3.25, 3.27 (1H each, both d, *J* = 11.5 Hz, H_2-15); ^{13}C NMR (CD_3OD, 125 MHz) δ_C: 124.6 (C-2), 110.6 (C-3), 119.3 (C-4), 119.8 (C-5), 122.4 (C-6), 112.2 (C-7), 138.0 (C-8), 128.9 (C-9), 28.5 (C-10), 54.9 (C-11), 174.9 (C-12), 168.8 (C-14), 41.8 (C-15), 171.5 (C-16); HR-ESI-MS: *m/z* 303.09854 $[M – H]^–$ (calcd for $C_{14}H_{13}O_5N_2$, 303.09755).

Equisetinine A (**9**)

White powder; $[\alpha]_D^{25}$ –111.4 (*cocn* 0.10, in MeOH); CD (*conc* 0.001 M, MeOH) mdeg (λ_{nm}) : +1.63 (283), –8.56 (254), +10.84 (231), –9.97 (201); ^{1}H NMR (CD_3OD, 500 MHz) δ_H: 5.73 (1H, s, H-2), 4.99 (1H, d, *J* = 9.0 Hz, H-9), [2.44 (1H, dd, *J* = 9.0, 14.0 Hz), 2.73 (1H, br. d, *ca.* *J* = 14 Hz), H_2-5], 7.43 (1H, br. d, *ca.* *J* = 8 Hz, H-8), 7.19 (1H, t like, *ca.* *J* = 8 Hz, H-9), 7.34 (1H, t like, *ca.* *J* = 8 Hz, H-10), 7.79 (1H, br. d, *ca.* *J* = 8 Hz, H-11), 3.07 (1H, s, Hβ-2'), nd (1H, Hα-2'), 3.80 (3H, s, 13-COOCH_3); ^{1}H NMR (DMSO-d_6, 500 MHz) δ_H: 5.75 (1H, s, H-2), 4.89 (1H, br. d, *ca.* *J* = 8 Hz, H-9), [2.43 (1H, dd, *J* = 9.0, 14.0 Hz), 2.58 (1H, br. d, *ca.* *J* = 14 Hz), H_2-5], 7.44 (1H, br. d, *ca.* *J* = 8 Hz, H-8), 7.17 (2H, t

like, *ca.* J = 8 Hz, H-9), 7.36 (1H, t like, *ca.* J = 8 Hz, H-10), 7.72 (1H, br. d, *ca.* J = 8 Hz, H-11), 2.91, 4.31 (1H each, both d, J = 16.5 Hz, H_2-2'), 3.72 (3H, s, 13-COOCH_3), 6.50 (1H, br. s, 6-OH); ^{13}C NMR (CD_3OD, 125 MHz) δ_C: 84.9 (C-2), 61.4 (C-4), 45.1 (C-5), 88.1 (C-6), 135.7 (C-7), 124.8 (C-8), 126.5 (C-9), 131.1 (C-10), 116.7 (C-11), 140.6 (C-12), 172.7 (C-13), 165.2 (C-1'), 46.0 (C-2'), 167.9 (C-3'), 53.3 (13-COOCH_3); ^{13}C NMR (DMSO-d_6, 125 MHz) δ_C: 82.8 (C-2), 59.5 (C-4), 43.7 (C-5), 86.2 (C-6), 134.7 (C-7), 123.8 (C-8), 124.6 (C-9), 129.6 (C-10), 114.6 (C-11), 139.0 (C-12), 170.9 (C-13), 163.3 (C-1'), 45.4 (C-2'), 165.4 (C-3'), 52.3 (13-COOCH_3); HR-ESI-MS: *m*/*z* 301.08301 [M – H]$^-$ (calcd for $C_{15}H_{13}O_5N_2$, 301.08972).

N-benzoylaspartate (**10**)

White powder; CD (*conc* 0.001 M, MeOH) mdeg (λ_{nm}): –2.41 (230), –0.38 (209), –1.14 (202), –0.92 (198); ^{1}H NMR (CD_3OD, 500 MHz) δ_H: 4.95 (1H, m, overlapped, H-2), [2.92 (1H, dd, J = 7.0, 16.5 Hz), 3.00 (1H, dd, J = 5.0, 16.5 Hz), H_2-3], 7.84 (2H, br. d, *ca.* J = 8 Hz, H-2',6'), 7.46 (2H, t like, *ca.* J = 8 Hz, H-3',5'), 7.54 (1H, t like, *ca.* J = 8 Hz, H-4'); ^{1}H NMR (DMSO-d_6, 500 MHz) δ_H: 4.71 (1H, m, overlapped, H-2), [2.83 (2H, dd, J = 7.0, 16.5 Hz), 2.83 (2H, dd, J = 7.0, 16.5 Hz), H_2-3], 7.85 (2H, br. d, *ca.* J = 8 Hz, H-2',6'), 7.48 (2H, t like, *ca.* J = 8 Hz, H-3',5'), 7.55 (1H, t like, *ca.* J = 8 Hz, H-4'); ^{13}C NMR (CD_3OD, 125 MHz) δ_C: 174.3 (C-1), 51.0 (C-2), 37.0 (C-3), 174.4 (C-4), 135.2 (C-1'), 128.4 (C-2',6'), 129.6 (C-3',5'), 132.9 (C-4'), 170.0 (C-7'); ^{13}C NMR (DMSO-d_6, 125 MHz) δ_C: 172.5 (C-1), 49.2 (C-2), 35.5 (C-3), 171.8 (C-4), 133.8 (C-1'), 127.2 (C-2',6'), 128.2 (C-3',5'), 131.3 (C-4'), 165.9 (C-7'). HR-ESI-MS: *m*/*z* 236.05597 [M – H]$^-$ (calcd for $C_{11}H_{10}O_5N$, 236.05535).

Nicotinic acid (**11**)

White powder; ^{1}H NMR (CD_3OD, 600 MHz) δ_H: 9.12 (1H, d, *J* = 1.8 Hz, H-2), 8.41 (1H, td like, *ca. J* = 2, 8 Hz, H-4), 7.56 (1H, dd, *J* = 4.8, 7.8 Hz, H-5), 8.72 (1H, dd, *J* = 1.8, 4.8 Hz, H-6); ^{13}C NMR (CD_3OD, 150 MHz) δ_C: 151.3 (C-2), 129.0 (C-3), 139.2 (C-4), 125.2 (C-5), 153.6 (C-6), 168.0 (C-7); HR-ESI-MS: *m/z* 122.02339 $[M - H]^-$ (calcd for $C_6H_4O_2N$, 122.02366).

Adenine (**12**)

White powder; ^{1}H NMR (DMSO-d_6, 600 MHz) δ_H: 8.12 (1H, s, H-2), 8.10 (1H, s, H-8), 12.74 (1H, br. s, H-9), 7.10 (2H, s, 6-NH_2); ^{13}C NMR (DMSO-d_6, 150 MHz) δ_C: 152.3 (C-2), 150.0 (C-4), 118.1 (C-5), 155.7 (C-6), 139.0 (C-8); HR-ESI-MS: *m/z* 134.04608 $[M - H]^-$ (calcd for $C_5H_4N_5$, 134.04612).

β-Adenosine (**13**)

White powder; ^{1}H NMR (DMSO-d_6, 500 MHz) δ_H: 8.15 (1H, s, H-2), 8.10 (1H, s, H-8), 5.89 (1H, d, *J* = 6.0 Hz, H-1'), 4.62 (1H, q like, *ca. J* = 6 Hz, H-2'), 4.15 (1H, br. s, H-3'), 3.98 (1H, m, H-4'), [3.57 (1H, dd, *J* = 6.0, 11.0 Hz), 3.69 (1H, br. d, *ca. J* = 11 Hz), H_2-5'], 7.38 (2H, s, 6-NH_2); ^{13}C NMR (DMSO-d_6, 125 MHz) δ_C: 152.4 (C-2), 149.0 (C-4), 119.3 (C-5), 156.1 (C-6), 139.9 (C-8), 87.9 (C-1'), 73.4 (C-2'), 70.6 (C-3'), 85.9 (C-4'), 61.6 (C-5'); HR-ESI-MS: *m/z* 268.10379 $[M + H]^+$ (calcd for $C_{10}H_{14}O_4N_5$, 268.10403).

Guanosine (**14**)

White powder; ^{1}H NMR (DMSO-d_6, 500 MHz) δ_H: 10.66 (1H, br. s, H-1), 7.93 (1H, s, H-8), 5.69 (1H, d, J = 6.0 Hz, H-1'), 4.39 (1H, q like, *ca.* J = 6 Hz, H-2'), 4.09 (1H, t like, *ca.* J = 4 Hz, H-3'), 3.87 (1H, q like, *ca.* J = 4 Hz, H-4'), [3.53 (1H, tq like, *ca.* J = 5, 11 Hz), 3.61 (1H, tq like, *ca.* J = 5, 11 Hz) H_2-5'], 6.49 (2H, br. s, 2-NH_2); ^{13}C NMR (DMSO-d_6, 125 MHz) δ_C: 153.6 (C-2), 151.2 (C-4), 116.6 (C-5), 156.6 (C-6), 135.4 (C-8), 86.3 (C-1'), 73.6 (C-2'), 70.3 (C-3'), 85.1 (C-4'), 61.3 (C-5'); HR-ESI-MS: *m/z* 282.08472 $[M - H]^-$ (calcd for $C_{10}H_{12}O_5N_5$, 282.09112).
